# Whole genome sequence analysis reveals limited diversity among *Clostridioides difficile* ribotype 027 and 078 isolates collected in 22 hospitals in Berlin and Brandenburg, Germany

**DOI:** 10.1186/s13756-025-01565-y

**Published:** 2025-05-28

**Authors:** Esther E. Dirks, Vanessa Pfiffer, Genevieve Sohl, Fabian K. Berger, Ina Friesen, Johannes Friesen, Ralf Ignatius, Johannes Elias, Alexander Mellmann, Mardjan Arvand

**Affiliations:** 1 Unit Hospital Hygiene, Infection Prevention and Control, Department for Infectious Diseases, Robert Koch Institute, Berlin, Germany; 2 Labor Berlin – Charité Vivantes GmbH, Berlin, Germany; 3 National Reference Center for Clostridioides Difficile, Homburg Branch, Homburg/Saar, Germany; 4 Institute of Virology, Saarland University Medical Center, Homburg/Saar, Germany; 5 National and WHO Supranational Reference Center for Mycobacteria, Research Center Borstel, Borstel, Germany; 6 Medizinisches Versorgungszentrum Labor 28 GmbH, Berlin, Germany; 7 Institute of Microbiology, Infectious Diseases and Immunology, Charité – University Berlin, Berlin, Germany; 8 DRK Kliniken Berlin Westend, Berlin, Germany; 9 HMU Health and Medical University GmbH, Potsdam, Germany; 10 Institute of Hygiene, University Hospital Muenster and National Reference Center for Clostridioides Difficile, Münster Branch, Münster, Germany; 11 Institute for Medical Microbiology and Hygiene, Department of Infectious Diseases, University of Heidelberg, Heidelberg, Germany

**Keywords:** *Clostridioides difficile*, Ribotype 027, Ribotype 078, *Clostridioides difficile* infection (CDI), Whole genome sequencing (WGS), Ribotyping, Core genome multilocus sequence typing (cgMLST)

## Abstract

**Background:**

*Clostridioides difficile* infections (CDI) present significant health risks and are among the most important nosocomial infections. Ribotype (RT) 027 poses a particular risk due to its proposed “hypervirulence”. Traditionally, *C. difficile* isolates are characterized using PCR-ribotyping. More recently, whole genome sequence (WGS) analysis is increasingly used, which may provide a higher discriminatory power. This study aimed to assess the distribution of different *C. difficile* RTs in hospitals in the Berlin-Brandenburg area, and to analyse the heterogeneity within isolates of different ribotypes using WGS.

**Methods:**

Between February 2020 and November 2021, stool samples from patients with laboratory-confirmed CDI were collected from 22 hospitals (approximately 13,900 beds) in Berlin and Brandenburg. Toxigenic isolates (*n* = 476) were further characterized by ribotyping, antibiotic susceptibility testing, toxinotyping, and core genome multilocus sequence typing (cgMLST).

**Results:**

Sixty-five different RTs were detected, with RT014 (16.1%), RT027 (12.8%), and RT001 (7.6%) being the most prevalent. RT027 isolates exhibited resistance to several antibiotics. Further, cgMLST analysis revealed very close genetic relatedness between RT027 isolates despite being epidemiologically unrelated. Similar findings of a monomorphic population were observed for RT078 isolates. In contrast, other RTs showed a heterogenic population structure.

**Conclusions:**

This study provides first insights into the distribution of *C. difficile* genotypes, corresponding antimicrobial resistance, and clonal relatedness using cgMLST, highlighting RT027 as the second most common genotype for the studied area. For the monomorphic RT027 and RT078 populations, new definitions of clonal relatedness might be necessary.

**Supplementary Information:**

The online version contains supplementary material available at 10.1186/s13756-025-01565-y.

## Background

*Clostridioides difficil*e is a major nosocomial pathogen worldwide, with *C. difficile* infections (CDI) being the most common cause of antibiotic-associated diarrhoea [1]. These infections constitute a significant health threat, especially to vulnerable groups, such as the elderly or patients with comorbidities. They also cause substantial economic costs with high recurrence and mortality rates [2]. CDI manifests itself in variable clinical forms from mild diarrhoea - the most common symptom - to pseudomembranous colitis, toxic megacolon, sepsis or even death [3]. CDI is principally mediated by two exotoxins: toxin A (encoded by *tcdA*), and toxin B (encoded by *tcdB*), which are, in some ribotypes (RTs), accompanied by the binary toxin (encoded by *cdtA* and *cdtB).* Of additional concern is the fact that CDI is one of the most common hospital-acquired infections (HAI) [4]. Antimicrobial resistance is known to promote the selection of certain *C. difficile* strains such as RT027 [5].

A significant increase in CDI was noted at the beginning of the 21st century [5]. This correlated with the emergence of RT027, which among others, is referred to as “hypervirulent” [6]. Increased toxin production, fluoroquinolone resistance, elevated sporulation frequency, causation of large outbreaks and severe courses of CDI have led to the postulation of hypervirulence for this RT [7]. Other RTs such as RT078 have also been linked to increased severity of disease [8]. For Germany, a marked increase in RT027 prevalence between 2007 and 2019 was noted [9]. However, little is known about the distribution and prevalence of different RTs in northeast Germany, in particular for the capital area of Berlin and for Brandenburg (the federal state surrounding Berlin).

In the past, ribotyping has been frequently used for genotyping of *C. difficile* isolates in the context of outbreak investigations or to assess the distribution of different RTs in a geographic region [10]. In recent years, core genome multilocus sequence typing (cgMLST) has been applied in outbreak investigations and research studies [11]. The cgMLST method is based on the principle of analysing differences in the sequence of selected genes or loci, which are non-repetitive and conserved among all members of a species, so-called core genes [12]. Compared to ribotyping, cgMLST exhibits a higher discriminatory power, the results can be shared and compared more easily between laboratories, and it also allows phylogenetic analyses. However, a study by Baktash et al. recently showed limited discriminatory power of cgMLST within certain RTs originating from outbreak and non-outbreak situations [11]. Proper knowledge of the distribution of circulating strains and the population structure of *C. difficile* in non-outbreak situations is crucial for understanding and interpreting typing results in outbreak situations. In particular, the relatedness of isolates of the same RT within a given geographic region is poorly understood, but crucial in order to evaluate possible transmission events using molecular methods exhibiting a high discriminatory power.

With cgMLST being often used for RT027 outbreak analysis, this multi-centre study aimed to analyse cgMLST patterns of different RTs, including RT027 in a non-outbreak situation, to determine the genotypic distribution of *C. difficile* on a population-based level covering approximately 60% of hospital beds in Berlin and 8% in Brandenburg at the time. Moreover, the occurrence, spread, antimicrobial susceptibility and genetic relatedness of *C. difficile* should be determined.

## Materials and methods

### Bacterial isolates

Faecal samples of hospitalized patients with a laboratory-confirmed diagnosis of CDI were collected in 22 hospitals (approximately 13,900 beds) in Berlin and Brandenburg from February 2020 to December 2021. The samples were allocated for diagnostic purposes and sent to the respective hospitals’ laboratories. Samples being tested positive for toxigenic *C. difficile* in the primary laboratory were forwarded to the Robert Koch Institute (RKI) for inclusion in this study. For this study, a case was defined as a patient of > 2 years of age with a laboratory-confirmed CDI and symptoms of CDI. Only one sample per case was evaluated. Patient samples were included in the analysis regardless of the severity of the disease. Pseudonymised information about the patients, including age, gender, symptoms, and clinical outcome, was recorded when possible.

At the RKI, *C. difficile* was grown on selective agar (CHROMID^®^*C. difficile* Agar, bioMérieux, Marcy-l’Étoile, France) after alcohol shock treatment of the stool samples as described previously [13]. Samples that were low in volume or produced no growth on selective media were also directly streaked on blood agar. Identification was performed using standard microbiological techniques and a latex agglutination test for *C. difficile* (Oxoid™ *Clostridium difficile*, Fisher Scientific, Schwerte, Germany). All isolates were tested for in vitro production of toxins A and B with ELISA (RIDASCREEN^®^C. difficile ToxinA/B, r-Biopharm, Darmstadt, Germany). Toxin-producing isolates were sent to the German National Reference Center (NRC) for *C. difficile* for ribotyping, toxin gene determination, cgMLST, and antibiotic susceptibility testing. Selected isolates that could not be assigned to a known RT in the NRC were sent to the Department of Medical Microbiology, University of Leiden, the Netherlands, for further ribotyping.

### PCR ribotyping and toxin gene detection

Capillary gel electrophoresis-based PCR ribotyping and toxin gene detection (*tcdA*, *tcdB*,* cdtA* and *cdtB*) were carried out as described before [10, 14]. For ribotyping, a standardized protocol of the European Society of Clinical Microbiology and Infectious Diseases was used [15].

### Whole genome sequencing and core genome multilocus sequence typing (cgMLST)

Genomic DNA of bacterial isolates were extracted using the Monarch^®^ Genomic DNA Purification Kit (New England Biolabs, Ipswich, MA, USA) as described in the manufacturer’s protocol. Whole genome sequencing was done either on an Illumina MiSeq^®^ system (Illumina Inc., San Diego, CA, USA) using the Nextera XT library preparation kit version 2 and the 250 bp-paired-end protocol (Illumina Inc.) or on a PacBio^®^ Sequel IIe system (Pacific Biosciences, Menlo Park, CA, USA) using the SMRTbell^®^ Express Template Prep Kit 2.0 (Pacific Biosciences Inc.). Illumina reads were *de novo*-assembled with the aid of the SKESA assembler with default parameters. PacBio sequencing data were assembled *de novo* using the SMRT^®^ Link software suite versions 10 or 11 with default parameters. For cgMLST, a previously published cgMLST scheme for *C. difficile* was used [16] and alleles were extracted and analysed using the SeqSphere^+^ software (Ridom GmbH, Münster, Germany) and a threshold of ≤ 6 allele differences for the maximum number of differing alleles for isolates that likely belonged to the same clone [16]. For graphical representations, either minimum-spanning or neighbour-joining trees were generated using SeqSphere^+^ and displayed using the same software or iToL [17].

### Antimicrobial susceptibility testing

Antibiotic susceptibility testing was performed as previously described [10, 14]. In short, epsilometry testing was used for metronidazole, vancomycin and moxifloxacin (Liofilchem, Roseto degli Abruzzi, Italy) and agar disk diffusion (Kirby Bauer method) for clarithromycin and rifampicin (Becton Dickinson, Heidelberg, Germany) on Columbia agar (Becton Dickinson) with a McFarland value of 4.0. For clarithromycin (15 µg disc) and rifampicin (5 µg disc), the complete lack of an inhibition zone was considered as “resistant”, in accordance with previous studies [10, 14]. EUCAST breakpoints or epidemiologic cut-off values (ECOFF) were used for epsilometry testing (v13.0 for metronidazole and vancomycin (breakpoints), v11.0 for moxifloxacin (ECOFF), https://www.eucast.org/clinical_breakpoints).

### Statistics

For statistical analysis, RStudio (Integrated Development for R. RStudio, PBC, Boston, MA) was used. A Shapiro-Wilk Test was utilized to determine whether the given dataset followed a normal distribution. Age group divergences and differences in cgMLST distance were analysed by a Mann-Whitney U test. The results were considered significant with a p-value < 0.05.

### Data availability

Whole genome sequencing data is deposited at NCBI Genbank under BioProject Accession Number PRJNA1260709.

## Results

Twenty-two hospitals (19 in Berlin and 3 in Brandenburg) participated in this study. During the study period, there were 88 hospitals in Berlin, indicating that the study was able to cover 22% of hospitals. As there were several tertiary care hospitals among the participating hospitals, the study was able to cover 12,350 of 20,636 (59.8%) hospital beds during this period. From Brandenburg, 3 of 63 (4.8%) hospitals participated in the study, which covered 1,203 of 15,225 (7.9%) of the total hospital beds in Brandenburg. The sample submission per hospital was heterogeneously distributed (range between 2 and 31; median 29 samples). Information on the ward from which the sample originated was available for 319 isolates. Of note, 110 (34.5%) samples stemmed from geriatric wards.

A total of 486 faecal samples from 486 patients with CDI were collected. Three patients and their respective samples were excluded because they did not meet the inclusion criteria. Fifty-four per cent of the patients were female and the median age was 79 years (range 6–95 years; mean 74.1 years). From the 483 samples, 496 *C. difficile* isolates could be retrieved. Of these, 476 isolates were toxigenic and subjected to further analysis.

**Fig. 1:**
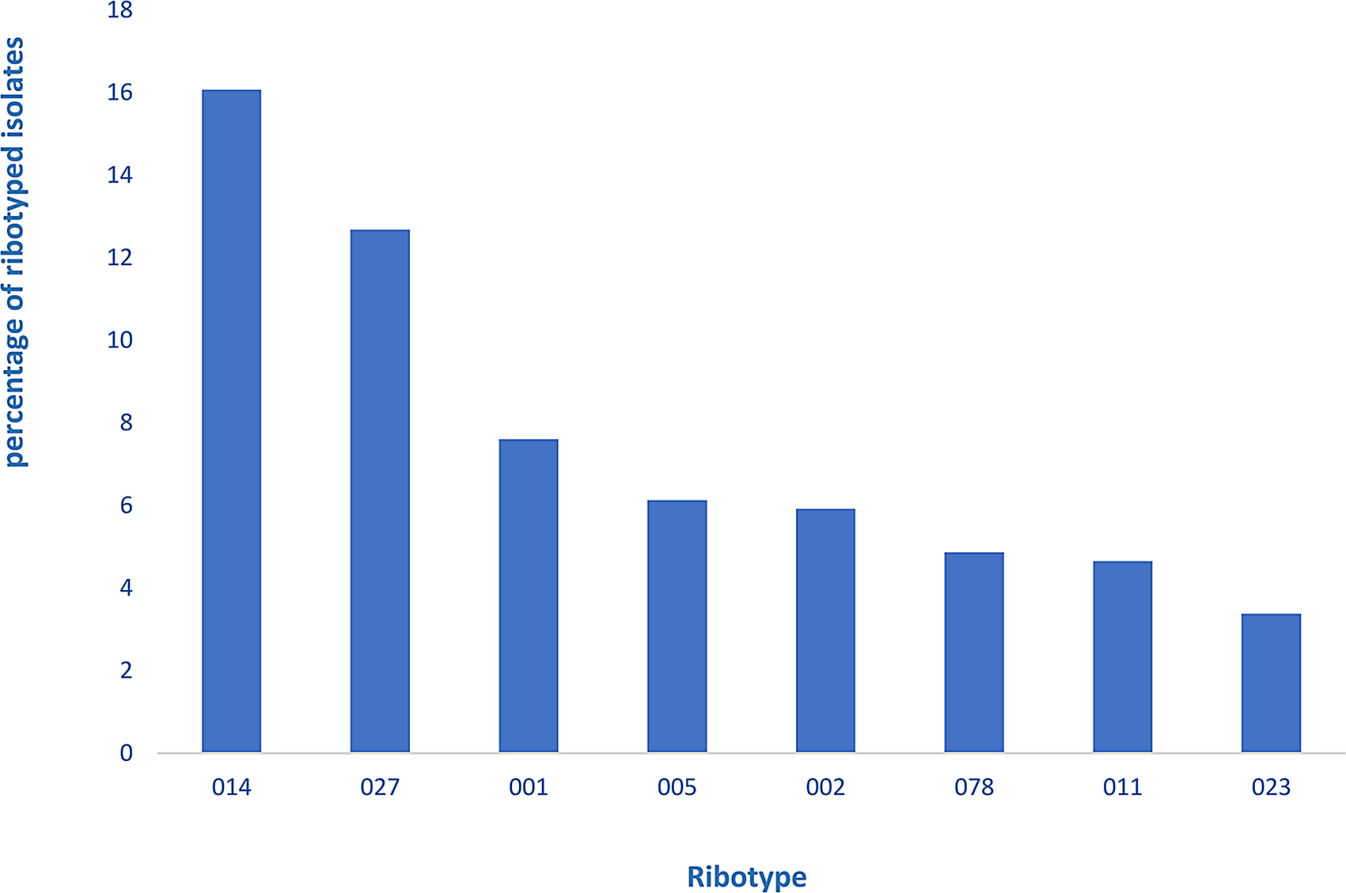
Percentage of the eight most commonly found ribotypes among the toxigenic isolates in this study (*n* = 476)

Among the 476 isolates, 65 different RTs were identified, while the RT could not be determined for seven isolates. The eight most frequent RTs were RT014 (16.1%), followed by RT027 (12.8%), RT001 (7.6%), RT005 (6.1%), RT002 (5.9%), RT078 (4.9%), RT011 (4.6%) and RT023 (3.4%) (Fig. 1). Patients infected with RT078 were significantly younger than the overall patient population (*p* = 0.014). The results for all isolates are presented in the Supplementary Table S1.

The distribution of different RTs in the participating hospitals varied. RT027 was present in all but one hospital from which ≥ 10 toxigenic isolates were retrieved. The proportion of RT027 among *C. difficile* isolates obtained from each hospital ranged from 0 to 38% (Supplementary Figure S1). The pattern of isolation of RT027 in the hospitals showed no evidence of temporal clustering in any of the participating hospitals (Supplementary Table S1).

The 476 toxin-positive isolates were analysed for the presence of toxin genes. Of these, 363 isolates harboured only *tcdA* and *tcdB*, whereas 113 isolates contained additionally the *cdtA* and *cdtB* genes. Seven different RTs were identified among the 113 isolates that harboured *cdtA* and *cdtB* (Table 1). All RT027 isolates carried *cdtA* and *cdtB*, as did all isolates assigned to RT023, RT045, RT078 and RT131 in this study.

**Table 1:** Ribotypes of *C. difficile* isolates harbouring the *cdtA* and *cdtB* gene (*n* = 113). One isolate of an unknown RT was also positive for *cdtA* and *cdtB*

Ribotype	Isolates harbouring cdtA/B *n* (%)
RT023 (*n* = 16)	16 (100)
RT027 (*n* = 61)	61 (100)
RT045 (*n* = 2)	2 (100)
RT078 (*n* = 23)	23 (100)
RT126 (*n* = 10)	9 (90)
RT127 (*n* = 2)	1 (50)
RT131 (*n* = 1)	1 (100)

Antimicrobial susceptibility testing was performed for 476 isolates (Table 2). No vancomycin resistance was detected. Resistance to at least one antibiotic was found in 158 of 476 isolates. The resistant isolates were assigned to 22 different RTs. In all RT027 isolates, antibiotic resistance was observed, and most of them were resistant to more than one of the antibiotics tested. One RT027 isolate showed resistance to metronidazole.

**Table 2:** Results of antimicrobial susceptibility testing of *C. difficile* isolates in correlation with ribotypes (RTs). Some isolates exhibit multiple resistances

Ribotype	Metronidazole	Clarithromycin	Rifampicin	Moxifloxacin	No Resistance *n* (%)
RT001 (*n* = 36)	0	17	0	17	0 (0)
RT002 (*n* = 28)	0	0	0	1	27 (96.4)
RT005 (*n* = 29)	0	1	0	1	27 (93.1)
RT006 (*n* = 1)	0	1	0	0	0 (0)
RT011 (*n* = 22)	0	6	0	3	16 (72.7)
RT012 (*n* = 11)	0	8	0	1	2 (18.2)
RT014 (*n* = 76)	0	1	0	12	63 (82.9)
RT015 (*n* = 13)	0	1	0	1	12 (92.3)
RT017 (*n* = 2)	0	2	0	1	0 (0)
RT023 (*n* = 16)	0	0	0	1	15 (93.8)
RT027 (*n* = 61)	1	59	57	59	0 (0)
RT045 (*n* = 2)	0	1	0	2	0 (0)
RT046 (*n* = 9)	0	6	0	0	3 (33.3)
RT078 (*n* = 23)	0	14	0	2	7 (30.4)
RT106 (*n* = 6)	0	3	0	2	2 (33.3)
RT126 (*n* = 10)	0	6	0	3	4 (40)
RT127 (*n* = 2)	0	0	0	1	1 (50)
RT216 (*n* = 2)	0	0	0	1	1 (50)
RT228 (*n* = 4)	0	0	0	1	3 (75)
RT328 (*n* = 1)	0	1	0	0	0 (0)
RT729 (*n* = 1)	0	1	0	0	0 (0)
RT808 (*n* = 1)	0	1	0	0	0 (0)

**Fig. 2:**
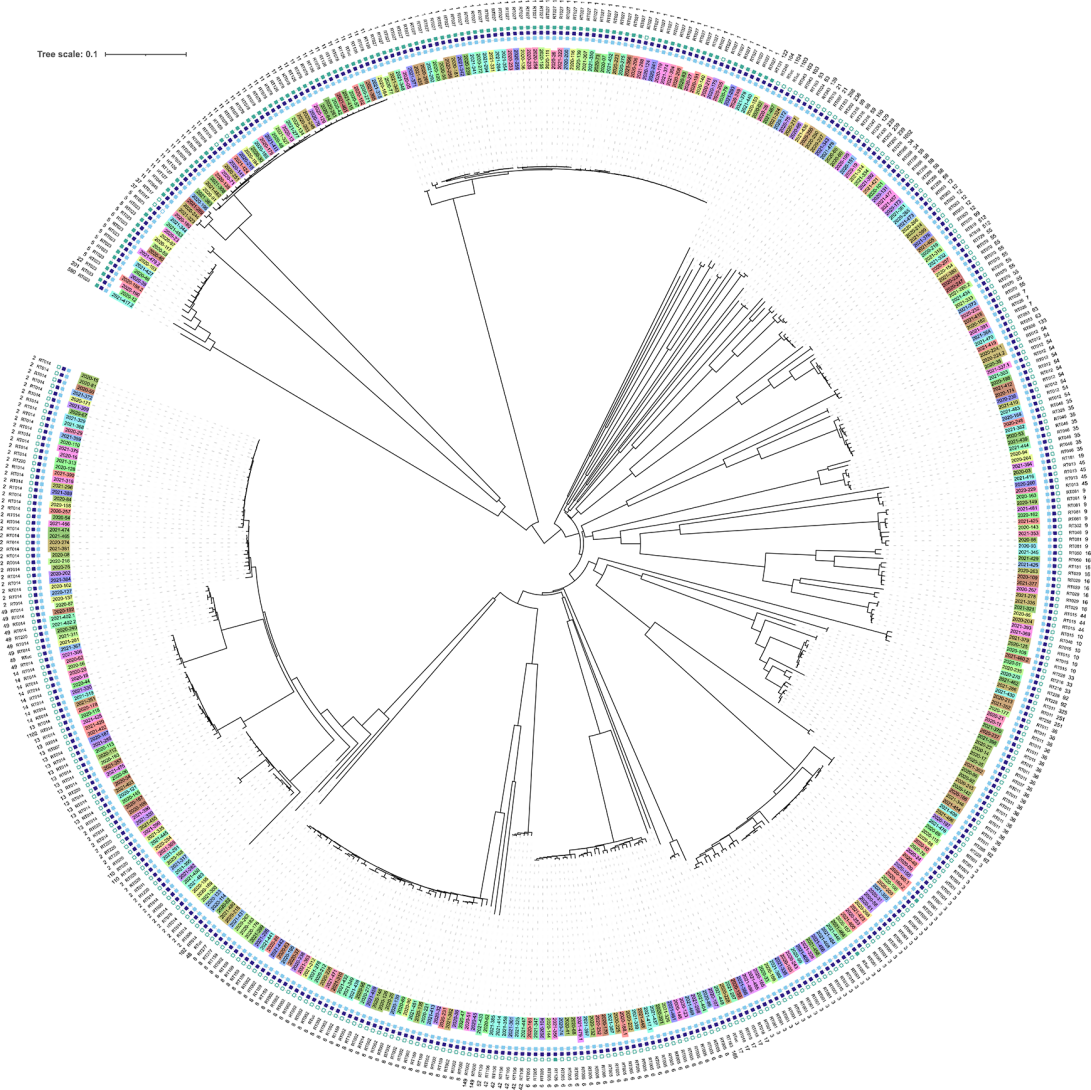
Neighbour-joining tree of all analysed toxigenic isolates based on cgMLST alleles. The leaves are coloured according to the hospital the isolate is derived from. In addition, the presence (complete boxes) of toxin genes (*tcdA* [light blue], *tcdB* [dark blue], *cdtA/B* [green]) and absence (empty boxes), RTs, and sequence types (STs) are given. RTuc, ribotype unclassified

**Fig. 3:**
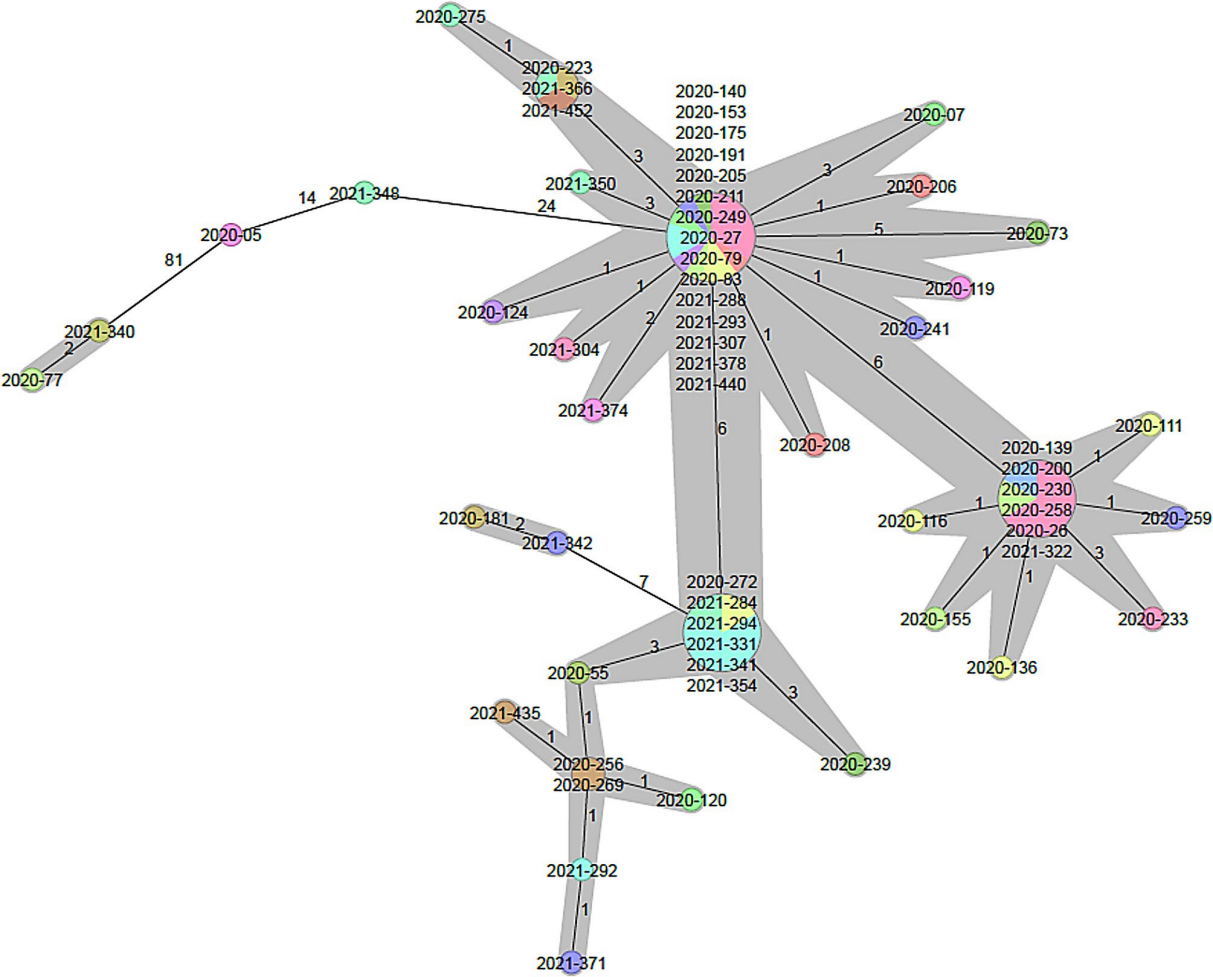
RT027 minimal spanning tree coloured according to hospital. The isolates belonging to a cluster are connected by grey bars. A cluster was defined with a threshold of ≤ 6 allele differences

**Fig. 4:**
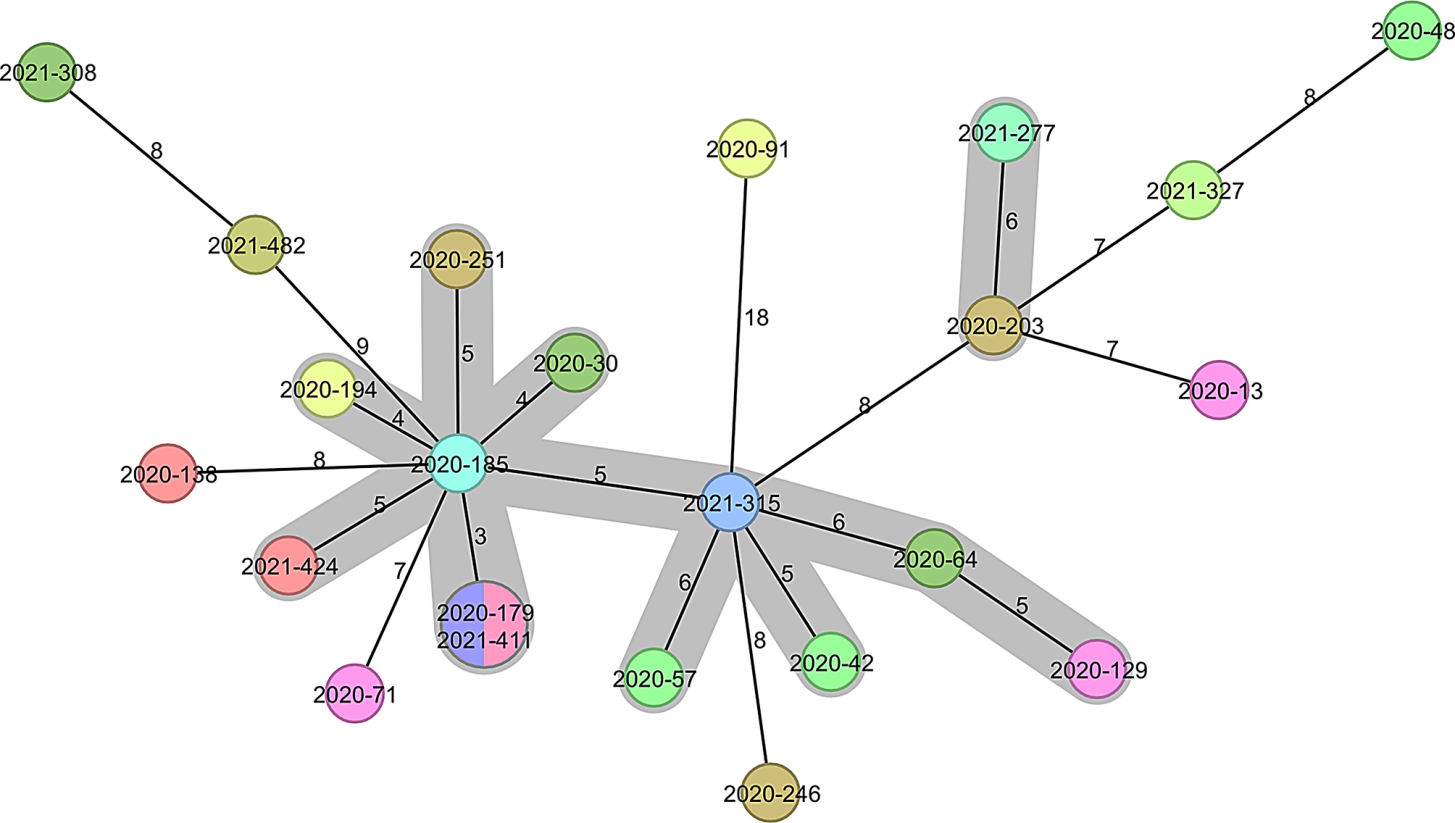
RT078 minimal spanning tree coloured according to the hospital the isolate derived from. The isolates belonging to a cluster are connected by grey bars. A cluster was defined with a threshold of ≤ 6 allele differences

Using cgMLST data and different modelling of phylogenetic trees, no clusters were found that could be assigned to specific hospitals (Fig. 2). The cgMLST results for RT027 and RT078 are depicted in Figs. 3 and 4, respectively. RT027 isolates formed a large cluster in which 55 of 61 isolates (90.2%) were represented. Only six RT027 isolates did not belong to this cluster. Similarly, most RT078 isolates were assigned to a large cluster containing 12 of 23 isolates (52.2%). For the RTs RT001, RT002, RT005, RT011, RT014 and RT023 the minimum spanning trees are shown in the supplementary Figures S2-S7.

The cgMLST distance matrixes for the eight most commonly found RTs were analysed and compared. For every RT-specific distance matrix, the mean and median distance, as well as the standard deviation, were calculated (Table 3). Distances between RT027 isolates and the rest of the eight most common RTs were significantly lower (*p* = 0.001). Isolates belonging to RT078 showed significantly lower distances as well (*p* = 0.001).

**Table 3:** Analysis of cgMLST distance (in allelic differences) matrixes. For the eight most common ribotypes (RTs) minimum, maximum, mean and median distance in alleles as well as standard deviation (SD) were calculated. The p-value refers to the cgMLST distance within the ribotype compared to the distance of all isolates

Ribotype	minimum	maximum	mean	median	SD	*p*
RT001	0	121	62	70.5	31.3	0.863
RT002	0	495	57.4	28	116.8	0.073
RT005	0	605	73.6	41	140.5	1
RT011	0	1382	241.4	182	352.6	1
RT014	0	1506	245	264.5	249.8	1
RT023	0	1956	283.3	48.5	608.5	1
RT027	0	106	14.6	8	22.9	0.001
RT078	0	24	10.7	10	4.7	0.001

## Discussion

This is the first systematic collection of data in this North-Eastern region of Germany to assess the RT distribution and corresponding antimicrobial resistance and the first study to perform a subsequent cgMLST analysis on isolates obtained from different hospitals in a non-outbreak situation in a defined period of time and area covering a substantial part of the population.

The RT most frequently detected was RT014 (16.1%), which is in line with earlier national and international studies that found RT014 among the most prevalent RTs in CDI patients [18, 19]. RT027 was the second most abundant genotype (12.8%) and was detected in all but one hospital from which ≥ 10 toxigenic isolates were retrieved. In a 5-year pan-European study from 2019, RT027 was the most abundant ribotype (12.6%), followed by RT001 (10.6%) and RT014 (7.8%) [5]. These results are comparable with our findings despite RT014 showing a slightly higher prevalence in our study. In a recent study conducted by the NRC, RT027 accounted for 3.5% of *C. difficile* isolates obtained from throughout Germany [10]. Our results show a markedly higher prevalence of RT027 in the Berlin-Brandenburg area. When looking at the level of individual hospitals, this study clearly shows that RT027 has become endemic and is rather common in hospitals in the Berlin-Brandenburg area.

Antibiotic resistance was mostly found against moxifloxacin, clarithromycin and rifampicin, and, as expected, a high percentage of RT027 isolates showed resistance against these antibiotics. Our findings of resistance patterns are in line with a study by Abdrabou et al. conducted at the same time. Here, the authors concluded that rifampicin and metronidazole resistance could be a driving force for RT027 selection and emphasised the importance of continuous surveillance efforts [14].

It has been shown that RT027 is not evenly distributed in different regions and even hospitals in Germany and Europe [9, 20, 21]. The proportion of RT027 in the hospitals included in our study varied markedly, ranging from 0 to 38%. This might be due to the fact that different types of hospitals contributed to this study. High percentages of RT027 were found in hospitals primarily caring for geriatric patients. Another reason could be the unnoticed nosocomial transmission of RT027 in some hospitals. However, our study design asked for the submission of isolates from non-outbreak situations from different wards and our data on the temporal pattern of isolation of RT027 in each hospital did not show any temporal clustering.

cgMLST revealed that the vast majority of RT027 isolates in this study belonged to one large cluster. We did not find any cluster that could be assigned to an individual hospital. Therefore, in our setting, cgMLST in conjunction with the cluster threshold definition given above would not allow for RT027 isolates to confirm transmission events within an individual hospital. In a study by Bakatash et al., the authors investigated a similar question when they compared the discriminatory power of cgMLST, whole-genome MLST, and single nucleotide polymorphism (SNP) analysis for individual RTs [11]. The authors concluded that specific cut-off thresholds and epidemiological data are necessary to recognize outbreaks of some specific RTs such as RT078. The results of our study suggest a similar situation for RT027. However, the authors of the latter paper also proposed a different cut-off value of three allelic differences for RT078. Currently, the literature lacks the average distance between RT027 isolates using cgMLST. In a recent study using cgMLST, it has been shown that the isolates with ST1/RT027 type cluster very tightly together and form a single homogenous cluster [22]. Similarly, it has been shown that ST11/RT078 isolates from different continents, countries and host species are very closely (clonally) related [23]. Taken together, there is increasing evidence that the vast majority of RT027 isolates, as well as RT078, are clonally related to each other. This is in line with our results showing a close clonal relationship between epidemiologically unrelated isolates within the latter RTs, which is striking when compared to other genotypes. These findings agree with the fact that the epidemic RT027 and RT078 strains have evolved recently and showed a fast expenditure during the last two decades. In conclusion, the presented data suggests that cgMLST of the latter RTs is limited as a single indicator for nosocomial transmission in the context of outbreak investigations. Hence, the results of an analysis using this method should be interpreted only in combination with sound epidemiological data. Further studies using cgMLST are needed to determine the overall heterogeneity among isolates of these RTs in a diverse sample from different hospitals, areas and countries.

On the other hand, our findings of just three RT027 clusters and almost all isolates belonging to one major cluster (*n* = 55) could also indicate a clonal lineage circulating in the Berlin and Brandenburg area. Many patients in this study were geriatric patients; thus, they might likely have already stayed in other hospitals or came from long-term care facilities in the areas, where this specific RT027 lineage has become endemic. Not only could this lineage be circulating within hospitals but also in different communities that belong to the catchment area of the specific hospitals. Unfortunately, no data exist on the RT distribution in community settings in the same region and time.

The “hypervirulent” RT078 strain accounted for 4.8% of all toxigenic isolates, which is in accordance with a previous German-wide study [10]. Interestingly, similar to our findings for RT027, cgMLST revealed that most RT078 isolates collected in this study belong to one large cluster that was not associated with a specific hospital. RT078 is associated with livestock and food production, thus, showing a zoonotic potential [23]. This strain may cause severe symptoms in younger persons with fewer co-morbidities [24, 25]. Our findings align with recent studies that have reported the emergence of this strain [26, 27], which could be due to a change in selection pressure. RT078 shows resistance to tetracyclines, an antibiotic still used in the veterinary field, especially in livestock. Dingle et al. have postulated that the intensive use of tetracyclines in agriculture and the subsequent selective pressure, as well as the fast spread of the pathogen via an international food chain, could be the reason for the spread of RT078 in humans [26]. Whether or not this could translate to the emergence of RT078 in healthcare should be addressed in future investigations.

The RT023 prevalence of 3.4% in this investigation is similar to a recent study [10] and should be of concern since this genotype is linked to more severe clinical outcomes [28]. Similar prevalence rates have been noted in Denmark, the Netherlands and Great Britain [29]. Due to the fact that RT023 might form atypical white colonies on selective agar, it might also be possibly missed in cultural diagnostics [28]. Interestingly, four out of sixteen RT023 isolates in this study were detected in the respective samples together with another toxigenic isolate indicating a coinfection with two RTs.

This study has some limitations. The data were collected during the COVID-19 pandemic, which could have had an impact on the number of samples collected or on the composition of the patient groups that were enrolled. It is important to note that the participation was voluntary, and the resources of the hospitals and laboratories were limited particularly due to this situation. The study is further limited by the sample size and coverage of hospitals participating in the Brandenburg area.

Our study demonstrates the need for molecular CDI surveillance on local, national and international levels. If pursued by a follow-up study, the presented data could serve as a basis for investigating the evolving molecular epidemiology of *C. difficile* in this region. As also suggested by Persson et al., it could be advantageous to use cgMLST not only to investigate possible outbreaks and transmission events but also for national surveillance [30]. Its recent inclusion into the miGenomeSurv network (http://www.miGenomeSurv.org) is one step in this direction. Further studies are needed to better understand the evolution and population structure of epidemic strains, in particular RT027 and RT078.

In conclusion, our study shows that the epidemic RT027 strain is endemic and widespread in hospitals in the Berlin-Brandenburg area. It may help to increase the awareness of healthcare workers and improve compliance with infection prevention and control recommendations and antibiotic stewardship to combat the further spread of nosocomial *C. difficile* strains. Further investigations are required to assess the changing molecular epidemiology of this pathogen. The inclusion of a larger area and sample size could help to refine cluster definitions and the possible presence of area-specific circulating lineages.

## Electronic supplementary material

Below is the link to the electronic supplementary material.

Supplementary Material 1

## Data Availability

All Sequence data are available at NCBI GenBank under the bioproject accession number PRJNA1260709.

## References

[1] Lawson PA, Citron DM, Tyrrell KL, Finegold SM. Reclassification of *Clostridium difficile* as *Clostridioides difficile* (Hall and O’Toole 1935) Prévot 1938. Anaerobe. 2016;40:95– 9. 10.1016/j.anaerobe.2016.06.008. 10.1016/j.anaerobe.2016.06.008 27370902

[2] Reigadas Ramírez E, Bouza ES. Economic burden of *Clostridium difficile* infection in European countries. Adv Exp Med Biol. 2018;1050:1–12. 10.1007/978-3-319-72799-8_1. 29383660 10.1007/978-3-319-72799-8_1

[3] Abt MC, McKenney PT, Pamer EG. *Clostridium difficile* colitis: pathogenesis and host defence. Nat Rev Microbiol. 2016;14(10):609–20. 10.1038/nrmicro.2016.108. 27573580 10.1038/nrmicro.2016.108 PMC5109054

[4] Czepiel J, Dróżdż M, Pituch H, Kuijper EJ, Perucki W, Mielimonka A, et al. *Clostridium difficile* infection: review. Eur J Clin Microbiol Infect Dis. 2019;38(7):1211–21. 10.1007/s10096-019-03539-6. 30945014 10.1007/s10096-019-03539-6 PMC6570665

[5] Freeman J, Bauer MP, Baines SD, Corver J, Fawley WN, Goorhuis B, et al. The changing epidemiology of *Clostridium difficile* infections. Clin Microbiol Rev. 2010;23(3):529–49. 10.1128/cmr.00082-09. 20610822 10.1128/CMR.00082-09 PMC2901659

[6] O’Connor JR, Johnson S, Gerding DN. *Clostridium difficile* infection caused by the epidemic BI/NAP1/027 strain. Gastroenterology. 2009;136(6):1913–24. 10.1053/j.gastro.2009.02.073. 19457419 10.1053/j.gastro.2009.02.073

[7] Loo VG, Poirier L, Miller MA, Oughton M, Libman MD, Michaud S, et al. A predominantly clonal multi-institutional outbreak of *Clostridium difficile*-associated diarrhea with high morbidity and mortality. N Engl J Med. 2005;353(23):2442–9. 10.1056/NEJMoa051639. 16322602 10.1056/NEJMoa051639

[8] Mitchell M, Nguyen SV, Macori G, Bolton D, McMullan G, Drudy D, et al. *Clostridioides difficile* as a potential pathogen of importance to one health: A review. Foodborne Pathog Dis. 2022;19(12):806–16. 10.1089/fpd.2022.0037. 36516404 10.1089/fpd.2022.0037

[9] Marujo V, Arvand M. The largely unnoticed spread of *Clostridioides difficile* PCR ribotype 027 in Germany after 2010. Infect Prev Pract. 2020;2(4):100102. 10.1016/j.infpip.2020.100102. 34368730 10.1016/j.infpip.2020.100102 PMC8336157

[10] Abdrabou AMM, Bischoff M, Mellmann A, von Müller L, Margardt L, Gärtner BC et al. Implementation of a *Clostridioides difficile* Sentinel surveillance system in Germany: first insights for 2019–2021. Anaerobe 2022:102548. 10.1016/j.anaerobe.2022.102548 10.1016/j.anaerobe.2022.102548 35307546

[11] Baktash A, Corver J, Harmanus C, Smits WK, Fawley W, Wilcox MH, et al. Comparison of Whole-Genome Sequence-Based methods and PCR ribotyping for subtyping of *Clostridioides difficile*. J Clin Microbiol. 2022;60(2):e0173721. 10.1128/jcm.01737-21. 34911367 10.1128/jcm.01737-21 PMC8849210

[12] Janezic S, Rupnik M. Development and implementation of whole genome Sequencing-Based typing schemes for *Clostridioides difficile*. Front Public Health. 2019;7:309. 10.3389/fpubh.2019.00309. 31709221 10.3389/fpubh.2019.00309 PMC6821651

[13] Arvand M, Moser V, Schwehn C, Bettge-Weller G, Hensgens MP, Kuijper EJ. High prevalence of *Clostridium difficile* colonization among nursing home residents in Hesse, Germany. PLoS ONE. 2012;7(1):e30183. 10.1371/journal.pone.0030183. 22253917 10.1371/journal.pone.0030183 PMC3256225

[14] Abdrabou AMM, Ul Habib Bajwa Z, Halfmann A, Mellmann A, Nimmesgern A, Margardt L, et al. Molecular epidemiology and antimicrobial resistance of *Clostridioides difficile* in Germany, 2014–2019. Int J Med Microbiol. 2021;311(4):151507. 10.1016/j.ijmm.2021.151507. 33915347 10.1016/j.ijmm.2021.151507

[15] Fawley WN, Knetsch CW, MacCannell DR, Harmanus C, Du T, Mulvey MR, et al. Development and validation of an internationally-standardized, high-resolution capillary gel-based electrophoresis PCR-ribotyping protocol for *Clostridium difficile*. PLoS ONE. 2015;10(2):e0118150. 10.1371/journal.pone.0118150. 25679978 10.1371/journal.pone.0118150 PMC4332677

[16] Bletz S, Janezic S, Harmsen D, Rupnik M, Mellmann A. Defining and evaluating a core genome multilocus sequence typing scheme for genome-Wide typing of *Clostridium difficile*. J Clin Microbiol. 2018;56(6):e01987–17. 10.1128/jcm.01987-17. 29618503 10.1128/JCM.01987-17 PMC5971537

[17] Letunic I, Bork P. Interactive tree of life (iTOL) v6: recent updates to the phylogenetic tree display and annotation tool. Nucleic Acids Res. 2024;52(W1):W78–82. 10.1093/nar/gkae268. 38613393 10.1093/nar/gkae268 PMC11223838

[18] Freeman J, Vernon J, Pilling S, Morris K, Nicolson S, Shearman S, et al. Five-year Pan-European, longitudinal surveillance of *Clostridium difficile* ribotype prevalence and antimicrobial resistance: the extended closer study. Eur J Clin Microbiol Infect Dis. 2020;39(1):169–77. 10.1007/s10096-019-03708-7. 31811507 10.1007/s10096-019-03708-7 PMC6962284

[19] Arvand M, Hauri AM, Zaiss NH, Witte W, Bettge-Weller G. Epidemiology of severe *Clostridium difficile* infections in Hesse, Germany in 2008–2009. Dtsch Med Wochenschr. 2010;135(40):1963–7. 10.1055/s-0030-1263342. 20922636 10.1055/s-0030-1263342

[20] Labbé AC, Poirier L, Maccannell D, Louie T, Savoie M, Béliveau C, et al. *Clostridium difficile* infections in a Canadian tertiary care hospital before and during a regional epidemic associated with the BI/NAP1/027 strain. Antimicrob Agents Chemother. 2008;52(9):3180–7. 10.1128/aac.00146-08. 18573937 10.1128/AAC.00146-08 PMC2533448

[21] Arvand M, Bettge-Weller G. *Clostridium difficile* ribotype 027 is not evenly distributed in Hesse. Ger Anaerobe. 2016;40:1–4. 10.1016/j.anaerobe.2016.04.006. 10.1016/j.anaerobe.2016.04.006 27063988

[22] Wang YY, Xie L, Zhang WZ, Du XL, Li WG, Bia LL, et al. Application of a core genome sequence typing (cgMLST) pipeline for surveillance of *Clostridioides difficile* in China. Front Cell Infect Microbiol. 2023;13:1109153. 10.3389/fcimb.2023.1109153. 36992688 10.3389/fcimb.2023.1109153 PMC10040748

[23] Knight DR, Kullin B, Androga GO, Barbut F, Eckert C, Johnson S, et al. Evolutionary and genomic insights into clostridioides difficile sequence type 11: a diverse zoonotic and Antimicrobial-Resistant lineage of global. One Health Importance mBio. 2019;10(2):e00446–19. 10.1128/mBio.00446-19. 30992351 10.1128/mBio.00446-19 PMC6469969

[24] Goorhuis A, Bakker D, Corver J, Debast SB, Harmanus C, Notermans DW, et al. Emergence of *Clostridium difficile* infection due to a new hypervirulent strain, polymerase chain reaction ribotype 078. Clin Infect Dis. 2008;47(9):1162–70. 10.1086/592257. 18808358 10.1086/592257

[25] Walker AS, Eyre DW, Wyllie DH, Dingle KE, Griffiths D, Shine B, et al. Relationship between bacterial strain type, host biomarkers, and mortality in *Clostridium difficile* infection. Clin Infect Dis. 2013;56(11):1589–600. 10.1093/cid/cit127. 23463640 10.1093/cid/cit127 PMC3641870

[26] Dingle KE, Didelot X, Quan TP, Eyre DW, Stoesser N, Marwick CA et al. A Role for Tetracycline Selection in Recent Evolution of Agriculture-Associated *Clostridium difficile* PCR Ribotype 078. mBio. 2019;10(2):e02790-18. 10.1128/mBio.02790-18 10.1128/mBio.02790-18 PMC6414706 30862754

[27] Rupnik M, Widmer A, Zimmermann O, Eckert C, Barbut F. Clostridium difficile, toxinotype V, ribotype 078, in animals and humans. J Clin Microbiol. 2008;46(6):2146. 10.1128/JCM.00598-08. 18417662 10.1128/JCM.00598-08 PMC2446824

[28] Shaw HA, Preston MD, Vendrik KEW, Cairns MD, Browne HP, Stabler RA, et al. The recent emergence of a highly related virulent *Clostridium difficile* clade with unique characteristics. Clin Microbiol Infect. 2020;26(4):492–8. 10.1016/j.cmi.2019.09.004. 31525517 10.1016/j.cmi.2019.09.004 PMC7167513

[29] Wilcox MH, Shetty N, Fawley WN, Shemko M, Coen P, Birtles A, et al. Changing epidemiology of *Clostridium difficile* infection following the introduction of a National Ribotyping-Based surveillance scheme in England. Clin Infect Dis. 2012;55(8):1056–63. 10.1093/cid/cis614. 22784871 10.1093/cid/cis614

[30] Persson S, Nielsen HL, Coia JE, Engberg J, Olesen BS, Engsbro AL, et al. Sentinel surveillance and epidemiology of *Clostridioides difficile* in Denmark, 2016 to 2019. Euro Surveill. 2022;27(49):2200244. 10.2807/1560-7917.Es.2022.27.49.2200244. 36695439 10.2807/1560-7917.ES.2022.27.49.2200244 PMC9732923

