# Supplementary material for: Whole genome sequence analysis reveals limited diversity among Clostridioides difficile ribotype 027 and 078 isolates collected in 22 hospitals in Berlin and Brandenburg, Germany
Source: Antimicrob Resist Infect Control. 2025 May 28;14:56. doi: 10.1186/s13756-025-01565-y (PMC12121276; doi:10.1186/s13756-025-01565-y)

Supplementary Material

**Supplementary Table S1:** Samples included in the study, including hospital code, date of sample collection, ribotypes and MLST sequence type. NA, not available.

| **Isolate ID** | **Hospital Code** | **Date of sample Collection** | **Ribotype** | **MLST ST** |
| --- | --- | --- | --- | --- |
| 2020-01 | B | 19.02.2022 | RT228 | 33 |
| 2020-02 | A | 18.02.2020 | RT131 | 122 |
| 2020-03 | A | 20.02.2020 | RT013 | 45 |
| 2020-05 | C | 20.02.2020 | RT027 | 1 |
| 2020-06 | B | 23.02.2020 | RT014 | 13 |
| 2020-07 | B | 22.02.2020 | RT027 | 1 |
| 2020-08 | A | 24.02.2020 | RT014 | 2 |
| 2020-09 | S | NA | RT001 | 3 |
| 2020-10 | R | 03.03.2020 | RT001 | 3 |
| 2020-11 | S | 02.03.2020 | RT011 | no growth for subtyping |
| 2020-12 | B | 02.03.2020 | RT023 | 201 |
| 2020-13 | C | 03.03.2020 | RT078 | 11 |
| 2020-14 | A | 06.03.2020 | RT011 | 36 |
| 2020-15 | A | 04.03.2020 | RT014 | no growth for subtyping |
| 2020-16 | C | 04.03.2020 | RT014 | no growth for subtyping |
| 2020-17 | A | 07.03.2020 | RT011 | 36 |
| 2020-18 | C | 06.03.2020 | RT014 | no growth for subtyping |
| 2020-19 | C | 08.03.2020 | RT430 | 239 |
| 2020-20 | S | 10.03.2020 | RT014 | 14 |
| 2020-21 | S | 11.03.2020 | RT011 | 36 |
| 2020-22 | A | 09.03.2020 | RT011 | 36 |
| 2020-23 | C | 15.03.2020 | RT023 | 5 |
| 2020-24 | C | 13.03.2020 | RT001 | 3 |
| 2020-25 | A | 14.03.2020 | RT002 | 8 |
| 2020-26 | S | 25.03.2020 | RT027 | 1 |
| 2020-27 | S | 25.03.2020 | RT027 | 1 |
| 2020-28 | S | 06.04.2020 | RT248 | 104 |
| 2020-29 | S | 06.04.2020 | RT014 | no growth for subtyping |
| 2020-30 | F | 14.04.2020 | RT078 | 11 |
| 2020-31 | D | 13.04.2020 | RT001 | 3 |
| 2020-32 | D | 12.04.2020 | RT002 | 8 |
| 2020-33 | B | 17.04.2020 | RT001 | 3 |
| 2020-34 | E | 18.04.2020 | RT014 | 13 |
| 2020-35 | A | 19.04.2020 | RT037 | 36 |
| 2020-36 | D | 15.04.2020 | RT002 | 8 |
| 2020-37 | E | 19.04.2020 | RT002 | 8 |
| 2020-38 | A | 22.04.2020 | RT012 | 54 |
| 2020-39 | D | 22.04.2020 | RT023 | 5 |
| 2020-40 | E | 22.04.2020 | RT023 | 5 |
| 2020-41 | A | 20.04.2020 | RT005 | 6 |
| 2020-42 | B | 20.04.2020 | RT078 | 11 |
| 2020-44 | B | 21.04.2020 | RT014 | 14 |
| 2020-45 | D | 22.04.2020 | RT002 | 149 |
| 2020-46 | B | 22.04.2020 | RT023 | 5 |
| 2020-47 | B | 23.04.2020 | RT002 | 8 |
| 2020-48 | B | 24.04.2020 | RT078 | 11 |
| 2020-49 | R | 26.04.2020 | RT001 | 3 |
| 2020-50 | E | 25.04.2020 | RT014 | no growth for subtyping |
| 2020-51 | C | 25.04.2020 | RT002 | 8 |
| 2020-52 | S | 11.05.2020 | RT014 | 14 |
| 2020-53 | F | 07.05.2020 | RT046 | 35 |
| 2020-54 | F | 07.05.2020 | RT014 | 2 |
| 2020-55 | A | 07.05.2020 | RT027 | 1 |
| 2020-56 | G | 07.05.2020 | RT014 | 14 |
| 2020-57 | B | 07.05.2020 | RT078 | 11 |
| 2020-58 | D | 08.05.2020 | RT001 | 3 |
| 2020-59 | G | 09.05.2020 | RT023 | no growth for subtyping |
| 2020-60 | A | 11.05.2020 | RT316 | 59 |
| 2020-61 | D | 11.05.2020 | RT001 | 3 |
| 2020-62 | B | 11.05.2020 | RT139 | 52 |
| 2020-63 | E | 10.05.2020 | RT159 | 8 |
| 2020-64 | F | 07.05.2020 | RT078 | 11 |
| 2020-65 | A | 15.05.2020 | RT316 | no growth for subtyping |
| 2020-66 | D | 17.05.2020 | RT024 | 53 |
| 2020-67 | F | 18.05.2020 | RT014 | 2 |
| 2020-68 | A | 18.05.2020 | RT005 | 6 |
| 2020-69 | B | 18.05.2020 | RT001 | 3 |
| 2020-70 | D | 20.05.2020 | RT126 | 11 |
| 2020-71 | C | 21.05.2020 | RT078 | 11 |
| 2020-73 | F | 25.05.2020 | RT027 | 1 |
| 2020-74 | H | 27.05.2020 | RT005 | 6 |
| 2020-75 | D | 04.06.2020 | RT043 | no growth for subtyping |
| 2020-76 | A | 05.06.2020 | RT014 | 2 |
| 2020-77 | G | 05.06.2020 | RT027 | 1 |
| 2020-78 | G | 06.06.2020 | RT001 | no growth for subtyping |
| 2020-79 | B | 07.06.2020 | RT027 | 1 |
| 2020-80 | B | 05.06.2020 | RT002 | 8 |
| 2020-81 | A | 06.06.2020 | RT014 | no growth for subtyping |
| 2020-82 | F | 06.06.2020 | RT014 | 102 |
| 2020-83 | F | 07.06.2020 | RT027 | no growth for subtyping |
| 2020-84 | A | 07.06.2020 | RT014 | 2 |
| 2020-85 | I | 08.06.2020 | RT015 | 44 |
| 2020-86 | A | 08.06.2020 | RT081 | 9 |
| 2020-87 | G | 08.06.2020 | RT014 | 49 |
| 2020-88 | R | 15.06.2020 | RT002 | 8 |
| 2020-89 | H | 19.06.2020 | RT005 | 6 |
| 2020-90 | I | 21.06.2020 | RT002 | 8 |
| 2020-91 | I | 21.06.2020 | RT078 | 11 |
| 2020-92 | A | 22.06.2020 | RT011 | 36 |
| 2020-93 | J | 26.06.2020 | RT081 | 9 |
| 2020-94 | I | 27.06.2020 | RT046 | 35 |
| 2020-95 | F | 28.06.2020 | RT002 | 8 |
| 2020-96 | A | 29.06.2020 | RT011 | 36 |
| 2020-97 | I | 29.06.2020 | RT023 | 5 |
| 2020-98 | I | 29.06.2020 | RT001 | 3 |
| 2020-99 | L | 29.06.2020 | RT001 | 3 |
| 2020-100 | G | 30.06.2020 | RT001 | 3 |
| 2020-101 | B | 03.07.2020 | RT056 | 34 |
| 2020-102 | I | 05.07.2020 | RT014 | 2 |
| 2020-103 | I | 06.07.2020 | RT023 | 5 |
| 2020-104 | D | 06.07.2020 | RT005 | 6 |
| 2020-105 | S | 09.07.2020 | RT001 | 3 |
| 2020-106 | E | 08.07.2020 | RT014 | 2 |
| 2020-107 | D | 07.07.2020 | RT163 | 185 |
| 2020-108 | B | 07.07.2020 | RT015 | 10 |
| 2020-109 | E | 07.07.2020 | RT029 | no growth for subtyping |
| 2020-110 | B | 09.07.2020 | RT014 | 2 |
| 2020-111 | I | 11.07.2020 | RT027 | 1 |
| 2020-112 | A | 09.07.2020 | RT014 | 13 |
| 2020-113 | B | 11.07.2020 | RT014 | 13 |
| 2020-114 | H | 10.07.2020 | RT006 | 2 |
| 2020-115 | G | 11.07.2020 | RT001 | 3 |
| 2020-116 | I | 12.07.2020 | RT027 | no growth for subtyping |
| 2020-117 | I | 13.07.2020 | RT023 | 5 |
| 2020-118 | B | 13.07.2020 | RT014 | 1102 |
| 2020-119 | C | 12.07.2020 | RT027 | 1 |
| 2020-120 | B | 13.07.2020 | RT027 | 1 |
| 2020-121 | L | 13.07.2020 | RT014 | 13 |
| 2020-123 | K | 12.07.2020 | RT005 | 6 |
| 2020-124 | D | 15.07.2020 | RT027 | no growth for subtyping |
| 2020-125 | A | 14.07.2020 | RT015 | 10 |
| 2020-126 | A | 16.07.2020 | RT002 | 8 |
| 2020-127 | H | 16.07.2020 | RT014 | 2 |
| 2020-128 | B | 16.07.2020 | RT220 | 2 |
| 2020-129 | C | 16.07.2020 | RT078 | 11 |
| 2020-130 | H | 17.07.2020 | RT002 | 8 |
| 2020-131 | D | 17.07.2020 | RT758 | 58 |
| 2020-132 | A | 15.07.2020 | RT005 | 6 |
| 2020-133 | H | 15.07.2020 | RT014 | 2 |
| 2020-134 | G | 16.07.2020 | RT126 | 11 |
| 2020-135 | A | 16.07.2020 | RT002 | 8 |
| 2020-136 | I | 16.07.2020 | RT027 | 1 |
| 2020-137 | I | 18.07.2020 | RT014 | 2 |
| 2020-138 | R | 23.07.2020 | RT078 | 11 |
| 2020-139 | G | 20.07.2020 | RT027 | 1 |
| 2020-140 | I | 21.07.2020 | RT027 | 1 |
| 2020-141 | D | 21.07.2020 | RT015 | 11 |
| 2020-142 | A | 23.07.2020 | RT011 | 36 |
| 2020-143 | G | 23.07.2020 | RT302 | 9 |
| 2020-144 | G | 23.07.2020 | RT005 | 6 |
| 2020-145 | B | 23.07.2020 | RT014 | 13 |
| 2020-146 | A | 25.07.2020 | RT014 | 8 |
| 2020-148 | C | 25.07.2020 | RT005 | 6 |
| 2020-149 | A | 26.07.2020 | RT081 | no growth for subtyping |
| 2020-150 | D | 27.07.2020 | RT001 | 3 |
| 2020-151 | H | 25.07.2020 | RT293 | 129 |
| 2020-152 | R | 01.08.2020 | RT126 | 11 |
| 2020-153 | I | 01.08.2020 | RT027 | 1 |
| 2020-154 | I | 01.08.2020 | RT070 | 55 |
| 2020-155 | G | 03.08.2020 | RT027 | 1 |
| 2020-156 | I | 04.08.2020 | RT076 | no growth for subtyping |
| 2020-157 | G | 04.08.2020 | RT001 | 3 |
| 2020-158 | I | 04.08.2020 | RT014 | 2 |
| 2020-159 | T | 05.08.2020 | RT018 | 17 |
| 2020-160.1 | R | 10.08.2020 | RT001 | 3 |
| 2020-160.2 | R | 11.08.2020 | RT023 | no growth for subtyping |
| 2020-161 | R | 11.08.2020 | RT005 | 6 |
| 2020-162 | B | 06.08.2020 | RT081 | 9 |
| 2020-163 | B | 07.08.2020 | RT081 | 9 |
| 2020-164 | H | 06.08.2020 | RT046 | 35 |
| 2020-165 | K | 05.08.2020 | RT001 | 3 |
| 2020-166 | E | 07.08.2020 | RT011 | 36 |
| 2020-167 | E | 08.08.2020 | RT020 | 2 |
| 2020-168 | G | 17.08.2020 | RT011 | 2 |
| 2020-169 | I | 14.08.2020 | RT014 | 2 |
| 2020-170 | E | 15.08.2020 | RT005 | 6 |
| 2020-171 | I | 16.08.2020 | RT014 | 2 |
| 2020-172 | H | 18.08.2020 | RT043 | 103 |
| 2020-173 | D | 18.08.2020 | RT258 | no growth for subtyping |
| 2020-174 | E | 17.08.2020 | RT012 | 54 |
| 2020-175 | D | 21.08.2020 | RT027 | 1 |
| 2020-176 | G | 22.08.2020 | RT159 | 8 |
| 2020-177 | G | 23.08.2020 | RT011 | 36 |
| 2020-178 | R | NA | RT014 | 13 |
| 2020-179 | S | 27.08.2020 | RT078 | 11 |
| 2020-180 | S | 04.09.2020 | RT017 | 37 |
| 2020-181 | O | 06.09.2020 | RT027 | 1 |
| 2020-182 | O | 05.09.2020 | RT026 | 7 |
| 2020-183 | G | 01.09.2020 | RT002 | 8 |
| 2020-184 | I | 06.09.2020 | RT126 | 11 |
| 2020-185 | L | 07.09.2020 | RT078 | 11 |
| 2020-186 | G | 08.09.2020 | RT001 | 3 |
| 2020-187 | M | 07.09.2020 | RT014 | 13 |
| 2020-188.1 | R | 07.09.2020 | RT005 | 6 |
| 2020-188.2 | R | 07.09.2020 | RT023 | 5 |
| 2020-190 | S | 11.09.2020 | RT023 | 22 |
| 2020-191 | R | 17.09.2020 | RT027 | 1 |
| 2020-192 | E | 27.09.2020 | RT014 | 49 |
| 2020-193 | G | 25.09.2020 | RT014 | 13 |
| 2020-194 | I | 25.09.2020 | RT078 | 11 |
| 2020-195 | H | 25.09.2020 | RT159 | 8 |
| 2020-196 | H | 25.09.2020 | RT127 | 11 |
| 2020-197 | D | 26.09.2020 | RT205 | 92 |
| 2020-198 | F | 21.09.2020 | RT012 | 54 |
| 2020-199 | K | 26.09.2020 | RT247 | no growth for subtyping |
| 2020-200 | H | 26.09.2020 | RT027 | 1 |
| 2020-201 | F | 26.09.2020 | RT005 | 6 |
| 2020-202 | D | 22.09.2020 | RT014 | 2 |
| 2020-203 | O | 25.09.2020 | RT078 | 11 |
| 2020-204 | O | 30.09.2020 | RT015 | 44 |
| 2020-205 | S | 21.09.2020 | RT027 | 1 |
| 2020-206 | R | 21.09.2020 | RT027 | no growth for subtyping |
| 2020-207 | R | 22.09.2020 | RT729 | 55 |
| 2020-208 | R | 23.09.2020 | RT027 | 1 |
| 2020-209 | U | 24.09.2020 | RT001 | 3 |
| 2020-211 | S | 02.10.2020 | RT027 | 1 |
| 2020-212 | P | 28.09.2020 | RT002 | 8 |
| 2020-213 | Q | 27.09.2020 | RT011 | 251 |
| 2020-214 | N | 30.09.2020 | RT003 | 12 |
| 2020-215 | M | 27.09.2020 | RT159 | 11 |
| 2020-216 | G | 29.09.2020 | RT014 | 2 |
| 2020-217 | O | 14.10.2020 | RT103 | 53 |
| 2020-218 | O | 16.10.2020 | RT277 | 8 |
| 2020-219 | P | 10.10.2020 | RT070 | 99 |
| 2020-221 | P | 21.10.2020 | RT159 | 8 |
| 2020-222 | O | 26.10.2020 | RT005 | 6 |
| 2020-223 | O | 31.10.2020 | RT027 | 1 |
| 2020-224.1 | O | 01.11.2020 | RT012 | 54 |
| 2020-224.2 | O | 01.11.2020 | RT012 | 54 |
| 2020-225 | O | 08.11.2020 | RT005 | 6 |
| 2020-226 | O | 06.11.2020 | RT002 | 8 |
| 2020-227 | O | 13.11.2020 | RT153 | 268 |
| 2020-228 | O | 13.11.2020 | RT018 | 17 |
| 2020-229 | S | 16.11.2020 | RT013 | 45 |
| 2020-230 | S | 16.11.2020 | RT027 | 1 |
| 2020-231 | R | 17.11.2020 | RT002 | 8 |
| 2020-232 | S | 14.11.2020 | RT070 | 55 |
| 2020-233 | S | 17.11.2020 | RT027 | 1 |
| 2020-234 | T | 10.11.2020 | RT070 | 55 |
| 2020-235 | G | 15.11.2020 | RT216 | 33 |
| 2020-236 | M | 15.11.2020 | RT012 | 54 |
| 2020-237 | E | 14.11.2020 | RT011 | 36 |
| 2020-238 | D | 15.11.2020 | RT002 | 8 |
| 2020-239 | F | 13.11.2020 | RT027 | 1 |
| 2020-240 | F | 13.11.2020 | RT220 | no growth for subtyping |
| 2020-241 | M | 18.11.2020 | RT027 | 1 |
| 2020-243 | C | 16.11.2020 | RT001 | 3 |
| 2020-244 | O | 23.11.2020 | RT045 | 11 |
| 2020-245 | O | 24.11.2020 | RT011 | 36 |
| 2020-246 | O | NA | RT078 | 11 |
| 2020-247 | T | 18.11.2020 | RT070 | 55 |
| 2020-248 | R | 25.11.2020 | RT328 | 35 |
| 2020-249 | S | 26.11.2020 | RT027 | 1 |
| 2020-250 | O | 26.11.2020 | RT097 | 21 |
| 2020-251 | O | 27.11.2020 | RT078 | 11 |
| 2020-252 | R | 07.12.2020 | RT005 | 6 |
| 2020-253 | S | 07.12.2020 | RT001 | 3 |
| 2020-254 | V | 07.12.2020 | RT154 | 110 |
| 2020-255 | Q | 10.12.2020 | RT015 | 21 |
| 2020-256 | Q | 07.12.2020 | RT027 | 1 |
| 2020-257 | R | 09.12.2020 | RT014 | 2 |
| 2020-258 | S | 10.12.2020 | RT027 | 1 |
| 2020-259 | M | 08.12.2020 | RT027 | 1 |
| 2020-260 | M | 08.12.2020 | RT013 | 45 |
| 2020-261 | F | 08.12.2020 | RT126 | 11 |
| 2020-262 | G | 07.12.2020 | RT159 | 8 |
| 2020-263 | N | 07.12.2020 | RT029 | 16 |
| 2020-264 | I | 07.12.2020 | RT046 | 35 |
| 2020-265 | J | 07.12.2020 | RT258 | 58 |
| 2020-266 | I | 07.12.2020 | RT003 | 12 |
| 2020-267 | C | 09.12.2020 | RT029 | 16 |
| 2020-268 | V | 11.12.2020 | RT001 | 3 |
| 2020-269 | Q | 16.12.2020 | RT027 | 1 |
| 2020-270 | P | 17.12.2020 | RT216 | 33 |
| 2020-271 | P | 17.12.2020 | unclassified | 11 |
| 2020-272 | P | 20.12.2020 | RT027 | 1 |
| 2020-273 | P | 22.12.2020 | RT159 | no growth for subtyping |
| 2020-274 | O | 24.12.2020 | RT014 | no growth for subtyping |
| 2020-275 | P | 24.12.2020 | RT027 | 1 |
| 2021-280.2 | G | 04.01.2021 | RT070 | 55 |
| 2021-276 | P | 29.12.2020 | RT002 | 8 |
| 2021-277 | P | 31.12.2020 | RT078 | 11 |
| 2021-278 | O | 01.01.2021 | RT029 | 16 |
| 2021-281 | I | 03.01.2021 | RT014 | 49 |
| 2021-282 | D | 04.01.2021 | RT014 | 2 |
| 2021-284 | L | 06.01.2021 | RT027 | 1 |
| 2021-285 | D | 05.01.2021 | RT014 | 13 |
| 2021-286 | O | 09.01.2021 | RT228 | 92 |
| 2021-287 | P | 10.01.2021 | RT018 | 17 |
| 2021-288 | S | 18.01.2021 | RT027 | 1 |
| 2021-289 | T | 13.01.2021 | RT127 | 11 |
| 2021-290 | C | 17.01.2021 | RT220 | 2 |
| 2021-291 | L | 17.01.2021 | RT020 | 2 |
| 2021-292 | L | 17.01.2021 | RT027 | 1 |
| 2021-293 | M | 17.01.2021 | RT027 | 1 |
| 2021-294 | L | NA | RT027 | 1 |
| 2021-295 | M | 19.01.2021 | RT159 | 8 |
| 2021-296 | N | NA | RT014 | 2 |
| 2021-298 | D | 18.01.2021 | RT005 | 6 |
| 2021-299 | F | 18.01.2021 | RT159 | 8 |
| 2021-300 | G | 18.01.2021 | RT014 | 2 |
| 2021-301 | H | 20.01.2021 | RT106 | 42 |
| 2021-302 | Q | 19.01.2021 | RT011 | 36 |
| 2021-303 | P | 19.01.2021 | RT012 | 54 |
| 2021-304 | S | 02.02.2021 | RT027 | 1 |
| 2021-305 | S | 02.02.2021 | RT020 | 2 |
| 2021-306 | K | 22.01.2021 | RT014 | 49 |
| 2021-307 | G | 22.01.2021 | RT027 | 1 |
| 2021-308 | F | 26.01.2021 | RT078 | 11 |
| 2021-309 | D | 26.01.2021 | RT014 | 2 |
| 2021-310 | E | NA | RT002 | 8 |
| 2021-311 | G | 19.01.2021 | RT014 | 49 |
| 2021-312 | I | 21.01.2021 | RT002 | 8 |
| 2021-313 | B | 20.01.2021 | RT014 | 2 |
| 2021-314 | I | 20.01.2021 | RT202 | 239 |
| 2021-315 | H | 21.02.2021 | RT078 | 11 |
| 2021-316 | C | 21.01.2021 | RT014 | 2 |
| 2021-317 | H | 22.01.2021 | RT220 | 2 |
| 2021-318 | G | 26.02.2021 | RT819 | 512 |
| 2021-319 | L | 04.02.2021 | RT014 | 14 |
| 2021-320 | D | 05.02.2021 | RT220 | 2 |
| 2021-321 | F | 05.02.2021 | RT015 | 44 |
| 2021-322 | S | 08.02.2021 | RT027 | 1 |
| 2021-323 | O | 30.01.2021 | RT045 | 11 |
| 2021-324 | O | 05.02.2021 | unclassified | 1103 |
| 2021-325 | L | 15.02.2021 | RT106 | 42 |
| 2021-326 | G | 15.02.2021 | RT005 | 6 |
| 2021-327 | G | 16.02.2021 | RT078 | 11 |
| 2021-328 | K | 18.02.2021 | RT001 | 3 |
| 2021-329 | J | 19.02.2021 | RT014 | 2 |
| 2021-330 | D | 19.02.2021 | RT014 | 14 |
| 2021-331 | I | 19.02.2021 | RT027 | 1 |
| 2021-332 | L | 19.02.2021 | RT819 | 512 |
| 2021-333 | G | 19.02.2021 | RT070 | 55 |
| 2021-334 | G | 19.02.2021 | RT202 | 239 |
| 2021-335 | I | 19.02.2021 | RT020 | 2 |
| 2021-336 | O | 19.02.2021 | RT029 | 16 |
| 2021-337.1 | C | 27.02.2021 | RT012 | 54 |
| 2021-338 | M | 01.03.2021 | RT001 | 3 |
| 2021-339 | L | 27.02.2021 | RT005 | 6 |
| 2021-340 | N | NA | RT027 | 1 |
| 2021-341 | L | 02.03.2021 | RT027 | 1 |
| 2021-342 | M | 02.03.2021 | RT027 | 1 |
| 2021-343 | M | 02.03.2021 | RT202 | 236 |
| 2021-344 | J | 02.03.2021 | RT017 | 37 |
| 2021-345 | L | 26.02.2021 | RT050 | 16 |
| 2021-346 | O | 24.02.2021 | RT011 | 36 |
| 2021-347 | P | 26.02.2021 | RT005 | 6 |
| 2021-348 | P | 27.02.2021 | RT027 | 1 |
| 2021-349 | P | 01.03.2021 | RT002 | 8 |
| 2021-350 | P | 01.03.2021 | RT027 | 1 |
| 2021-351 | Q | 01.03.2021 | RT014 | 14 |
| 2021-352 | Q | 03.03.2021 | RT258 | 251 |
| 2021-353 | S | 10.03.2021 | RT046 | 9 |
| 2021-354 | L | 01.03.2021 | RT027 | 1 |
| 2021-355 | H | 04.03.2021 | RT001 | 3 |
| 2021-356 | C | 04.03.2021 | RT126 | 6 |
| 2021-357 | E | 08.03.2021 | RT014 | 13 |
| 2021-358 | L | 09.03.2021 | RT106 | 42 |
| 2021-359 | H | 09.03.2021 | RT014 | 2 |
| 2021-361 | L | 07.03.2021 | RT258 | 58 |
| 2021-362 | L | 07.03.2021 | RT046 | 35 |
| 2021-363 | S | 22.03.2021 | RT002 | 8 |
| 2021-364 | H | 13.03.2021 | RT053 | 63 |
| 2021-365 | E | 09.03.2021 | RT126 | 11 |
| 2021-366 | E | 13.03.2021 | RT027 | 1 |
| 2021-367 | H | 10.03.2021 | unclassified | 49 |
| 2021-368 | L | 10.03.2021 | RT014 | 2 |
| 2021-369 | C | 18.03.2021 | RT046 | 10 |
| 2021-370 | F | 15.03.2021 | RT011 | 36 |
| 2021-371 | M | 21.03.2021 | RT027 | 1 |
| 2021-372 | H | 20.03.2021 | RT014 | 2 |
| 2021-373 | H | 20.03.2021 | RT070 | 55 |
| 2021-374 | C | 02.04.2021 | RT027 | 1 |
| 2021-375 | C | 04.04.2021 | RT014 | 2 |
| 2021-376 | M | 03.04.2021 | RT003 | 12 |
| 2021-377 | E | 05.04.2021 | RT029 | 16 |
| 2021-378 | L | 06.04.2021 | RT027 | 1 |
| 2021-379 | O | 29.03.2021 | RT015 | 10 |
| 2021-380 | O | 28.03.2021 | RT070 | 55 |
| 2021-381 | O | 28.02.2021 | RT014 | 2 |
| 2021-382 | O | 18.04.2021 | RT159 | 8 |
| 2021-383 | F | 17.04.2021 | RT126 | 11 |
| 2021-384 | M | 21.04.2021 | RT014 | 2 |
| 2021-385 | L | 21.04.2021 | RT106 | 42 |
| 2021-386 | M | 20.04.2021 | RT018 | 17 |
| 2021-387 | L | 20.04.2021 | RT005 | 6 |
| 2021-388 | J | 22.04.2021 | RT001 | 3 |
| 2021-389 | M | 22.04.2021 | RT014 | 2 |
| 2021-390 | R | NA | RT014 | 2 |
| 2021-391 | C | 25.04.2021 | RT026 | 7 |
| 2021-392 | K | 26.04.2021 | RT076 | 1052 |
| 2021-393 | C | 27.04.2021 | RT015 | 10 |
| 2021-394 | K | 25.04.2021 | RT181 | 19 |
| 2021-395 | L | 27.04.2021 | RT014 | 2 |
| 2021-396 | K | 27.04.2021 | RT014 | 2 |
| 2021-397 | J | 29.04.2021 | RT005 | 6 |
| 2021-398 | F | 29.04.2021 | RT011 | 36 |
| 2021-399 | O | 01.05.2021 | RT003 | 12 |
| 2021-400 | R | 05.05.2021 | RT001 | 3 |
| 2021-401 | T | 07.05.2021 | RT126 | 11 |
| 2021-402.1 | P | 08.05.2021 | RT014 | 49 |
| 2021-402.2 | P | 08.05.2021 | RT014 | 49 |
| 2021-403 | Q | 07.05.2021 | RT014 | 13 |
| 2021-404 | P | 13.05.2021 | RT005 | 6 |
| 2021-405 | Q | 11.05.2021 | RT003 | 12 |
| 2021-406 | O | 19.05.2021 | RT011 | 36 |
| 2021-408 | L | 13.05.2021 | RT011 | 36 |
| 2021-409 | M | 18.05.2021 | RT001 | 3 |
| 2021-410 | N | 18.05.2021 | RT012 | 54 |
| 2021-411 | M | 25.05.2021 | RT078 | 11 |
| 2021-412 | E | 19.05.2021 | RT012 | 54 |
| 2021-413 | H | 19.05.2021 | RT159 | 8 |
| 2021-414 | L | 21.05.2021 | RT106 | 42 |
| 2021-415 | F | 21.05.2021 | RT126 | 11 |
| 2021-416 | L | 22.05.2021 | RT013 | 45 |
| 2021-417.1 | L | 23.05.2021 | RT005 | 6 |
| 2021-417.2 | L | 23.05.2021 | RT023 | 590 |
| 2021-418 | E | 25.05.2021 | RT026 | 7 |
| 2021-419 | R | 31.05.2021 | RT808 | 133 |
| 2021-420 | R | 01.06.2021 | RT014 | 13 |
| 2021-421 | U | 02.06.2021 | RT056 | 34 |
| 2021-422 | R | 04.06.2021 | RT007 | 13 |
| 2021-423 | R | 10.06.2021 | RT001 | 3 |
| 2021-424 | R | 14.06.2021 | RT078 | 11 |
| 2021-425 | R | 15.06.2021 | RT081 | 9 |
| 2021-426 | H | 09.06.2021 | RT181 | 16 |
| 2021-427 | J | 11.06.2021 | RT023 | 5 |
| 2021-428 | C | NA | RT014 | 13 |
| 2021-429 | F | 12.06.2021 | RT050 | 16 |
| 2021-430 | L | 10.06.2021 | RT011 | 325 |
| 2021-431 | H | 09.06.2021 | RT277 | 8 |
| 2021-432 | P | 08.06.2021 | RT002 | 8 |
| 2021-433 | P | 13.06.2021 | RT600 | 149 |
| 2021-434 | P | 14.06.2021 | RT070 | 55 |
| 2021-435 | Q | 17.06.2021 | RT027 | 1 |
| 2021-436 | V | 29.06.2021 | RT243 | 139 |
| 2021-437 | L | 12.06.2021 | RT005 | 6 |
| 2021-438 | L | 14.06.2021 | RT159 | 8 |
| 2021-439 | F | 13.06.2021 | RT046 | 35 |
| 2021-440 | L | 27.06.2021 | RT027 | 1 |
| 2021-441 | L | 28.06.2021 | RT159 | 8 |
| 2021-442 | M | 28.06.2021 | RT002 | 8 |
| 2021-444 | P | 26.06.2021 | RT046 | 35 |
| 2021-445 | P | 26.06.2021 | RT020 | 110 |
| 2021-446 | P | 27.06.2021 | RT001 | 3 |
| 2021-447 | P | 23.06.2021 | RT106 | 42 |
| 2021-448 | Q | 28.06.2021 | RT005 | 6 |
| 2021-449 | P | 05.08.2021 | unclassified | 17 |
| 2021-450 | P | 02.08.2021 | RT020 | 2 |
| 2021-451 | P | 02.08.2021 | RT002 | 8 |
| 2021-452 | P | 02.08.2021 | RT027 | 1 |
| 2021-453 | P | 06.08.2021 | RT023 | 5 |
| 2021-454 | Q | 09.08.2021 | RT011 | 36 |
| 2021-455 | N | 13.08.2021 | RT220 | 2 |
| 2021-456 | M | 19.08.2021 | RT015 | 3 |
| 2021-457 | C | 30.08.2021 | RT258 | 58 |
| 2021-458 | F | 30.08.2021 | RT015 | 3 |
| 2021-459 | M | 30.08.2021 | RT002 | 8 |
| 2021-460.2 | E | 01.09.2021 | RT015 | 10 |
| 2021-461 | M | 31.08.2021 | RT005 | 6 |
| 2021-462 | F | 28.08.2021 | RT228 | 92 |
| 2021-463 | L | 30.08.2021 | RT012 | 54 |
| 2021-464 | F | 29.08.2021 | RT001 | 3 |
| 2021-465 | F | 31.08.2021 | RT014 | 2 |
| 2021-466 | C | 17.09.2021 | RT014 | 2 |
| 2021-467 | F | 16.09.2021 | unclassified | 1104 |
| 2021-468 | H | 16.09.2021 | RT015 | 3 |
| 2021-469 | H | 18.09.2021 | RT005 | 6 |
| 2021-470 | L | 19.09.2021 | RT053 | 63 |
| 2021-471 | S | 04.10.2021 | unclassified | 8 |
| 2021-473 | H | 30.09.2021 | RT003 | 12 |
| 2021-474 | F | 03.10.2021 | RT014 | 2 |
| 2021-475 | K | 03.10.2021 | RT220 | 13 |
| 2021-476 | L | 07.10.2021 | RT228 | 92 |
| 2021-477 | C | 19.10.2021 | RT056 | 58 |
| 2021-478 | H | 18.10.2021 | RT316 | 59 |
| 2021-479.1 | K | 19.10.2021 | RT005 | 6 |
| 2021-479.2 | K | 19.10.2021 | RT023 | 5 |
| 2021-480 | N | 24.10.2021 | unclassified | 48 |
| 2021-481 | K | 23.10.2021 | RT081 | 9 |
| 2021-482 | N | 27.10.2021 | RT078 | 11 |
| 2021-483 | L | 25.11.2021 | RT014 | 2 |
| 2021-484 | H | 26.11.2021 | RT001 | 3 |
| 2021-485 | K | 28.11.2021 | RT001 | 3 |
| 2021-486 | K | 28.11.2021 | RT001 | 3 |

**Supplementary Table S2:** Samples from which two toxigenic Isolates could be retrieved due to differences in colony morphology.

| sample ID | ribotype isolate 1 | ribotype isolate 2 |
| --- | --- | --- |
| 2020-160 | RT001 | RT023 |
| 2020-188 | RT005 | RT023 |
| 2021-417 | RT005 | RT023 |
| 2021-479 | RT005 | RT023 |

**Supplementary Figure S1:** Proportion of RT027 among all *C. difficile* isolates obtained from a hospital. Hospitals from which 10 or more toxigenic isolates were retrieved (n=17) are shown.**
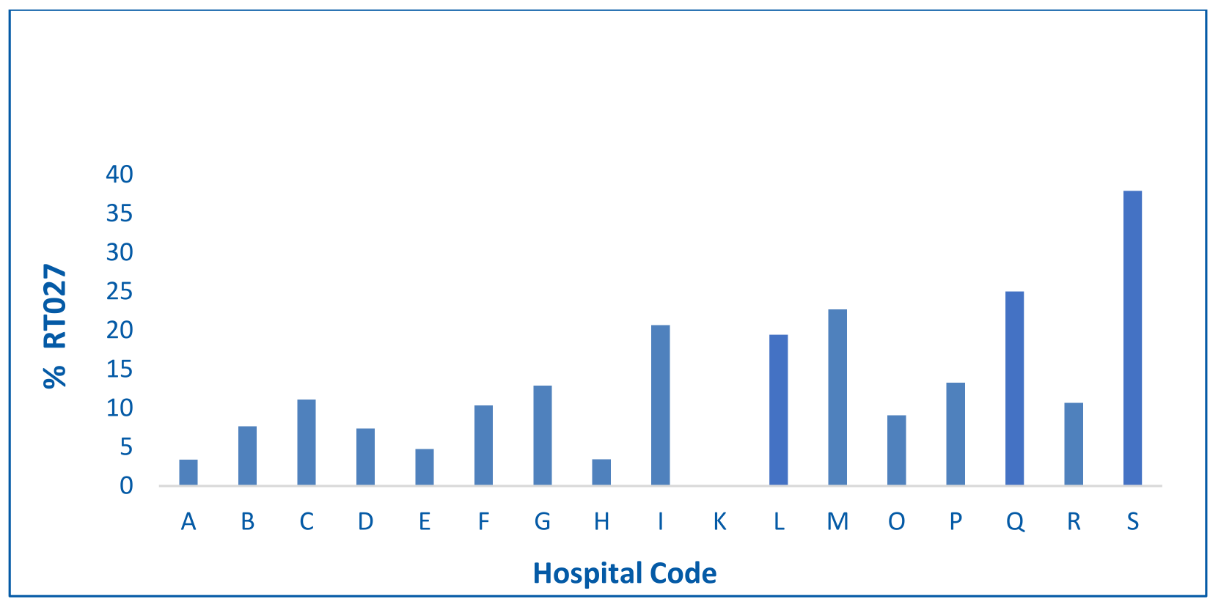
**

**Supplementary Figure S2:** RT001 minimal spanning tree coloured according to hospital. The isolates belonging to a cluster are connected by grey bars. A cluster was defined with a threshold of ≤ 6 allele differences.

**
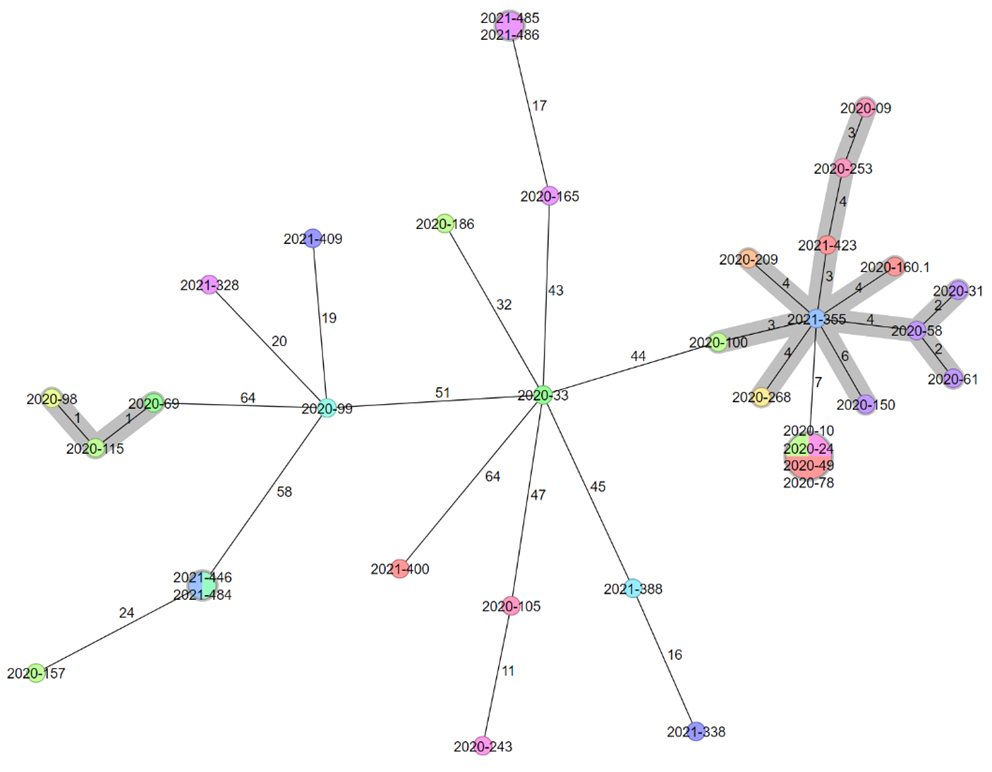
**

**Supplementary Figure S3:** RT002 minimal spanning tree coloured according to hospital. The isolates belonging to a cluster are connected by grey bars. A cluster was defined with a threshold of ≤ 6 allele differences.


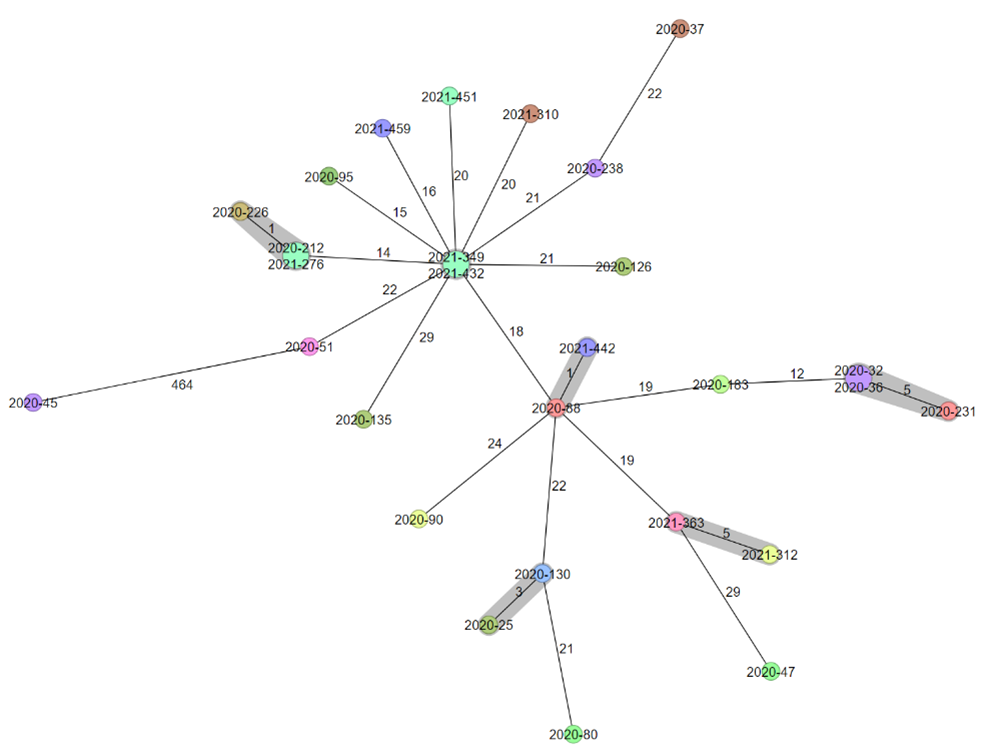


**Supplementary Figure S4:** RT005 minimal spanning tree coloured according to hospital. The isolates belonging to a cluster are connected by grey bars. A cluster was defined with a threshold of ≤ 6 allele differences.


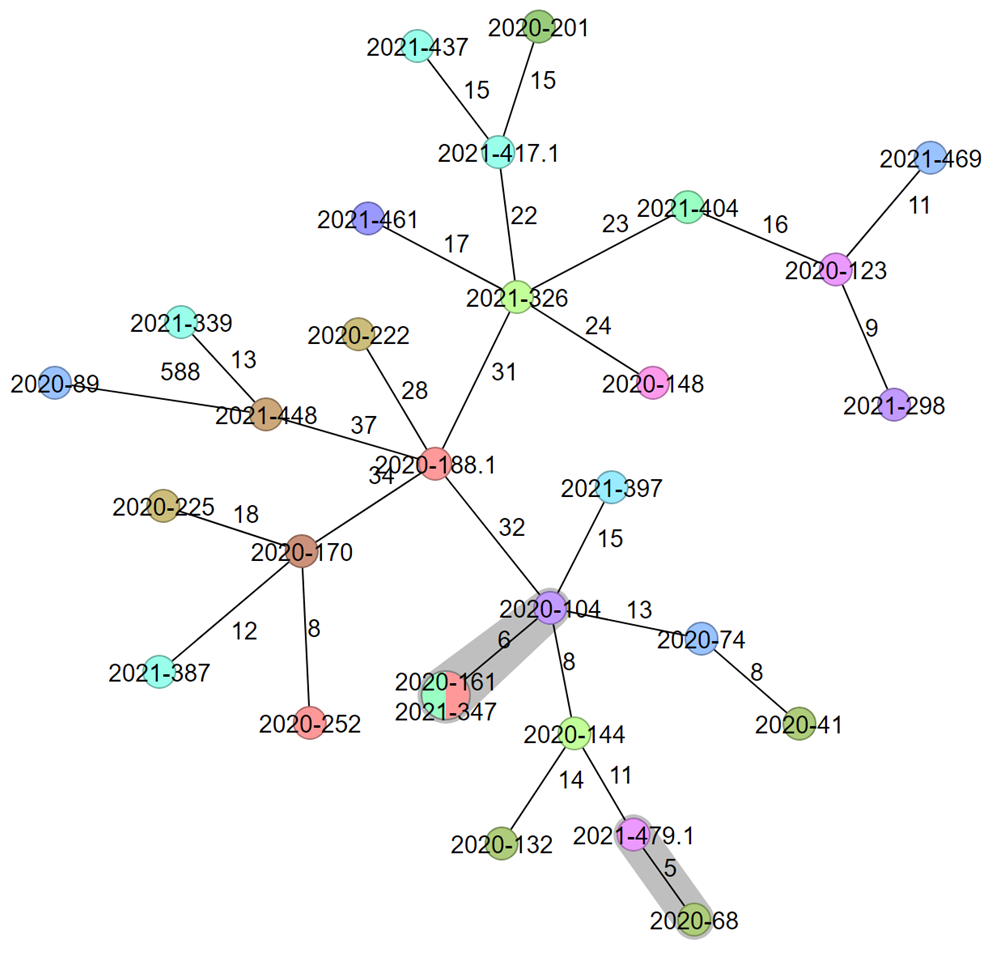


**Supplementary Figure S5:** RT011 minimal spanning tree coloured according to hospital. The isolates belonging to a cluster are connected by grey bars. A cluster was defined with a threshold of ≤ 6 allele differences.

**
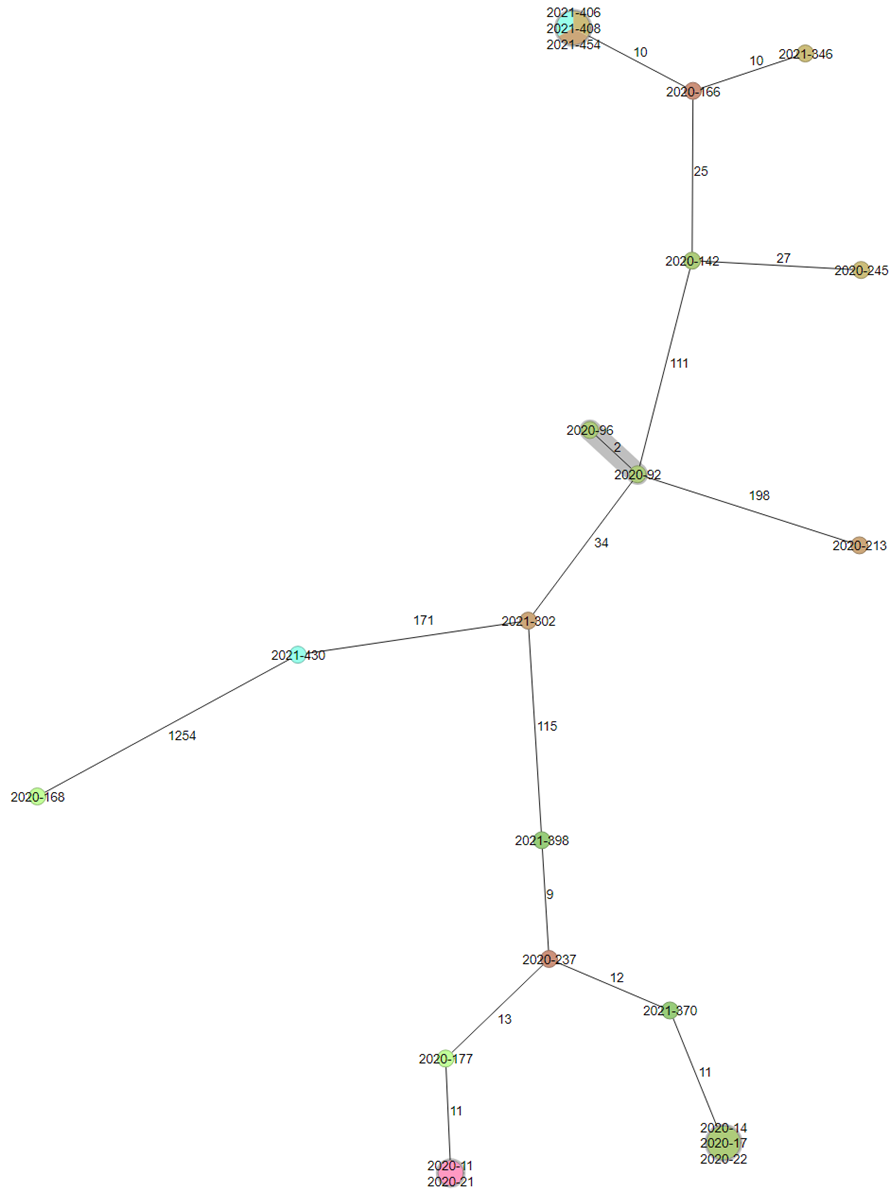
**

**Supplementary Figure S6:** RT014 minimal spanning tree coloured according to hospital. The isolates belonging to a cluster are connected by grey bars. A cluster was defined with a threshold of ≤ 6 allele differences.

**
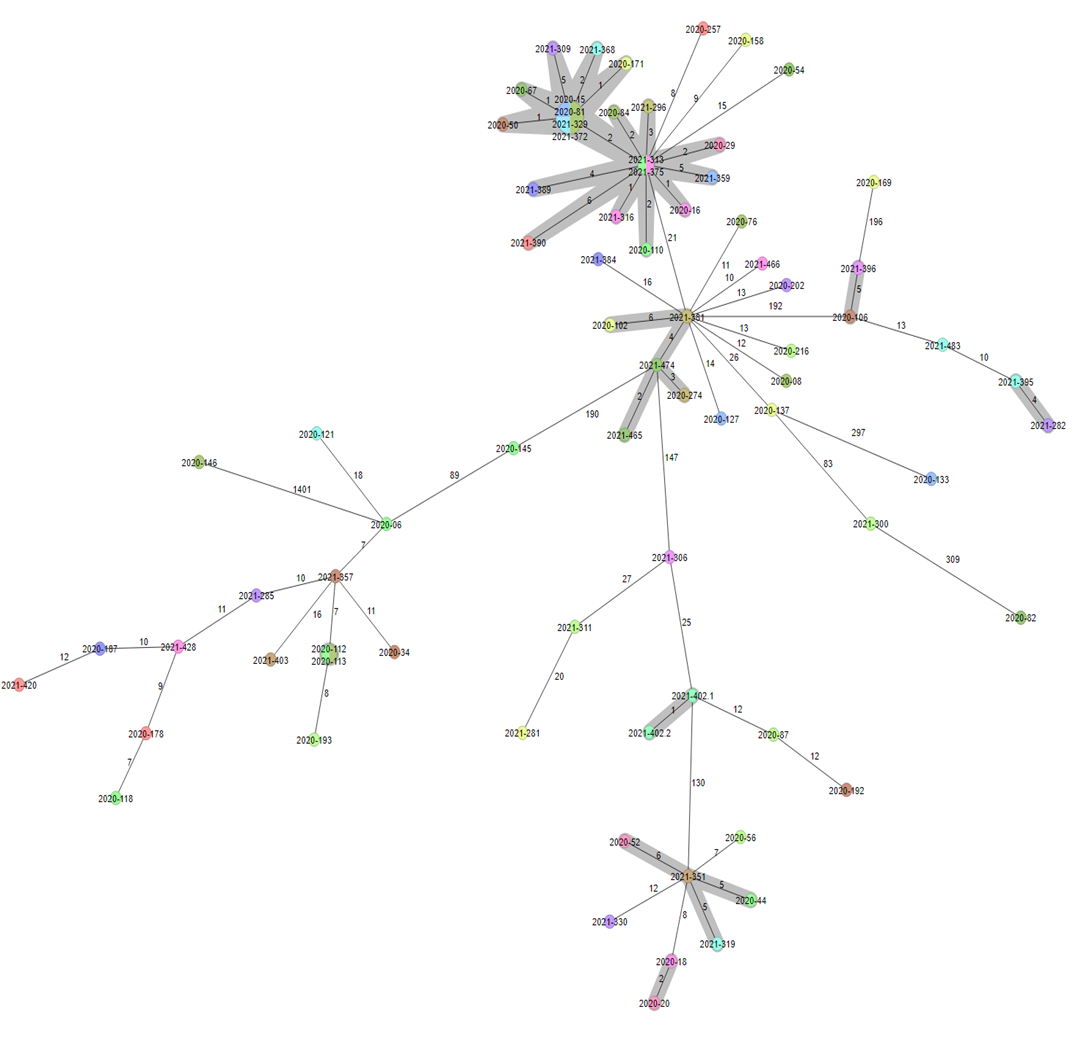
**

**Supplementary Figure S7:** RT023 minimal spanning tree coloured according to hospital. The isolates belonging to a cluster are connected by grey bars. A cluster was defined with a threshold of ≤ 6 allele differences.


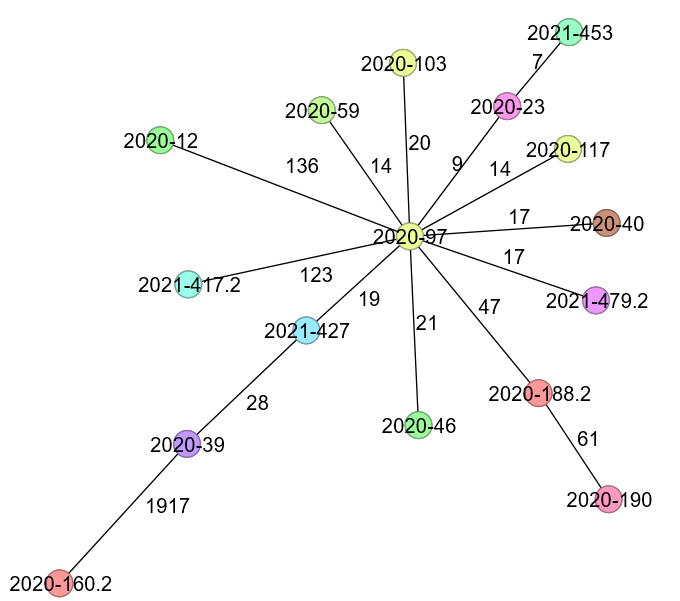

Supplement: Supplementary file 1 — Supplementary Material 1 [file 13756_2025_1565_MOESM1_ESM.docx]
